# Supplementary material for: Discriminative SKP2 Interactions with CDK-Cyclin Complexes Support a Cyclin A-Specific Role in p27KIP1 Degradation
Source: J Mol Biol. 2021 Mar 5;433(5):166795. doi: 10.1016/j.jmb.2020.166795 (PMC7895821; doi:10.1016/j.jmb.2020.166795)
Supplement: Supplementary data 1 [file mmc1.pdf]

Supplementary Information

Discriminative SKP2 interactions with CDK-cyclin complexes support a cyclin A-specific role in p27KIP1 degradation

Marco Salamina, Bailey C. Montefiore, Mengxi Liu, Daniel J. Wood, Richard Heath, James R. Ault, Lan-Zhen Wang, Svitlana Korolchuk, Arnaud Baslé, Martyna W. Pastok, Judith Reeks, Natalie J. Tatum, Frank Sobott, Stefan T. Arold, Michele Pagano, Martin E.M. Noble and Jane A. Endicott

Running title: A cyclin A-specific role in p27 degradation

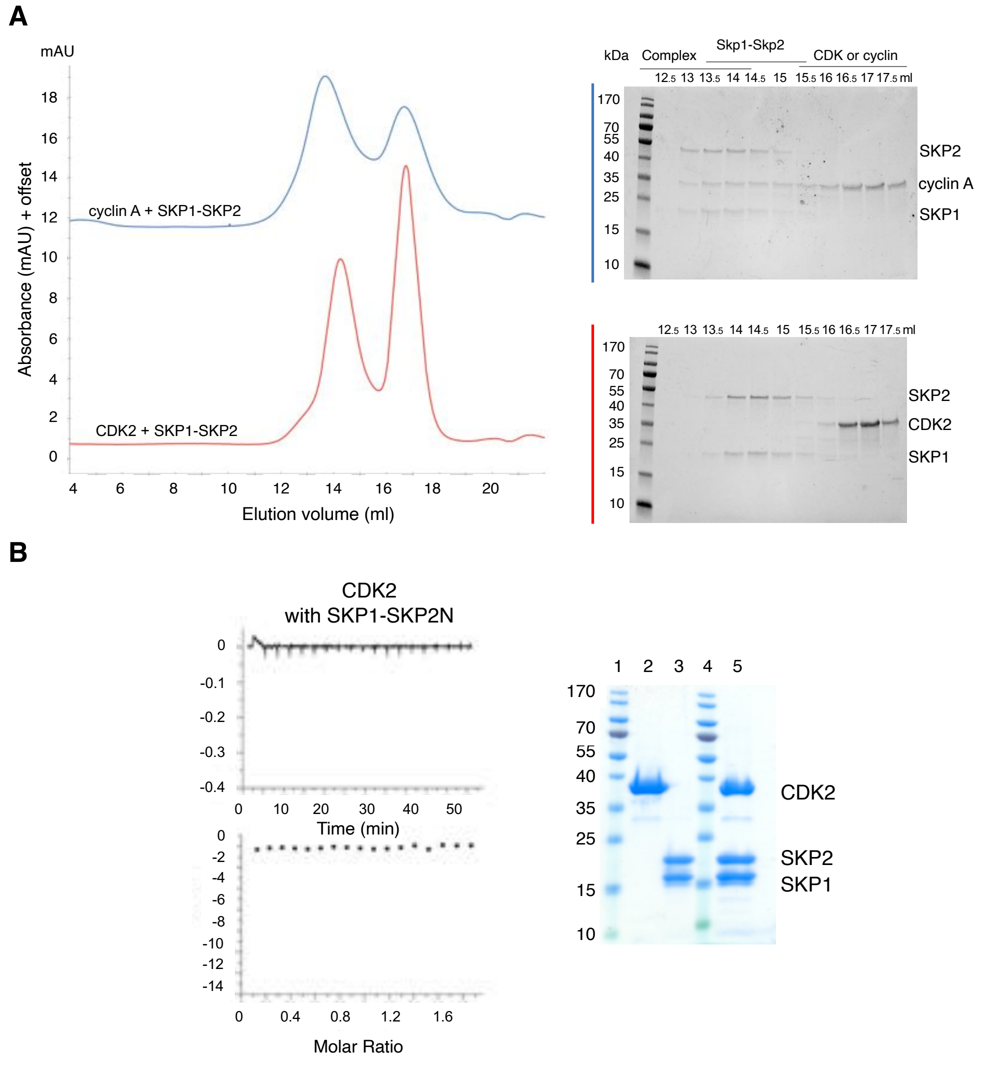

Supplementary Figure S1. Characterization of the binding between SKP1-SKP2 and CDK2 and cyclin A. Related to Figure 1.

(**A**) SKP1-SKP2 binds to cyclin A but not CDK2. SKP1-SKP2 was mixed with an excess of cyclin A (blue trace) or CDK2 (red trace) and analyzed by analytical size-exclusion chromatography. For each run comparable 0.5 ml fractions were analyzed by SDS-PAGE and visualized by InstantBlue staining. Chromatograms are to the same scale but have been offset on the y-axis to aid comparison. Cyclin A construct is bovine cyclin A residues 174-432. (**B**) SKP1-SKP2N does not bind to CDK2. Isothermal titration calorimetry (ITC) to measure the affinity of SKP1-SKP2N for monomeric CDK2. SDS-PAGE analysis confirms that post titration of SKP1-SKP2N and CDK2 all three proteins are intact (compare lanes 2 and 3 with lane 5). ITC reaction conditions are provided in Table 1. Chromatogram is representative of two experiments carried out using independently prepared proteins. The titration of monomeric CDK2 against SKP1-SKP2N was carried out once**.**

A

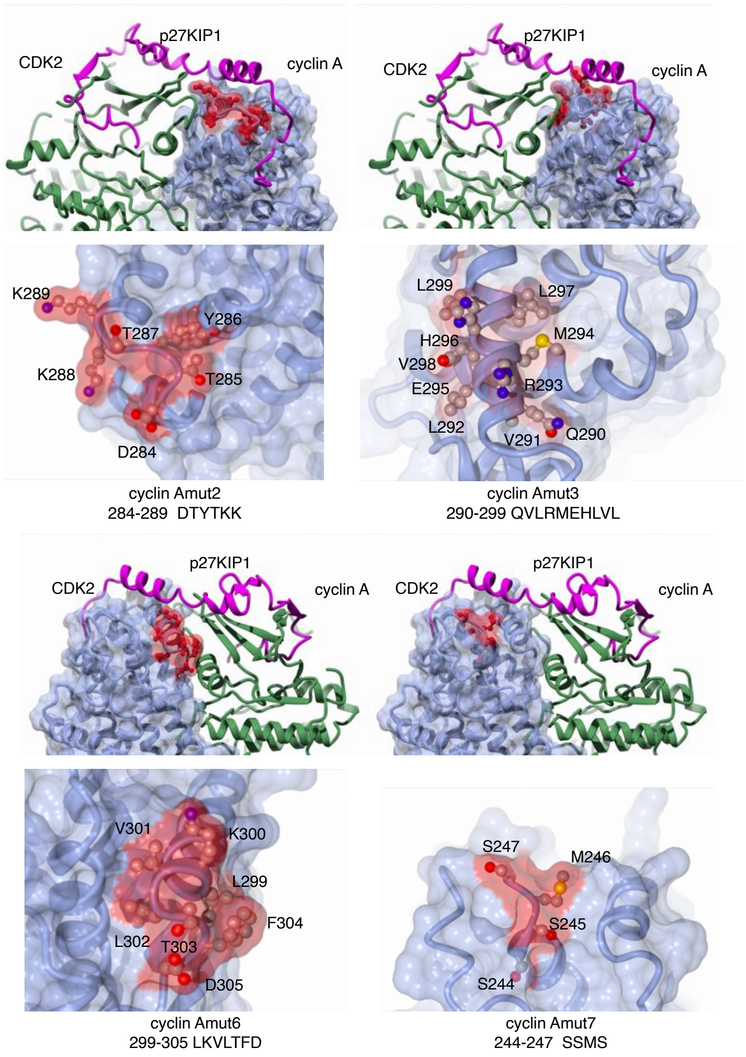
CDK2
p27KIP1
cyclin A
K289
T287
Y286
K288
T285
D284
cyclin Amut2
284-289 DTYTKK
L299
L297
H296
M294
V298
E295
R293
L292
V291
Q290
cyclin Amut3
290-299 QVLRMEHLVL
V301
K300
L299
F304
L302
T303
D305
cyclin Amut6
299-305 LKVLTFD
S247
M246
S245
S244
cyclin Amut7
244-247 SSMS

B

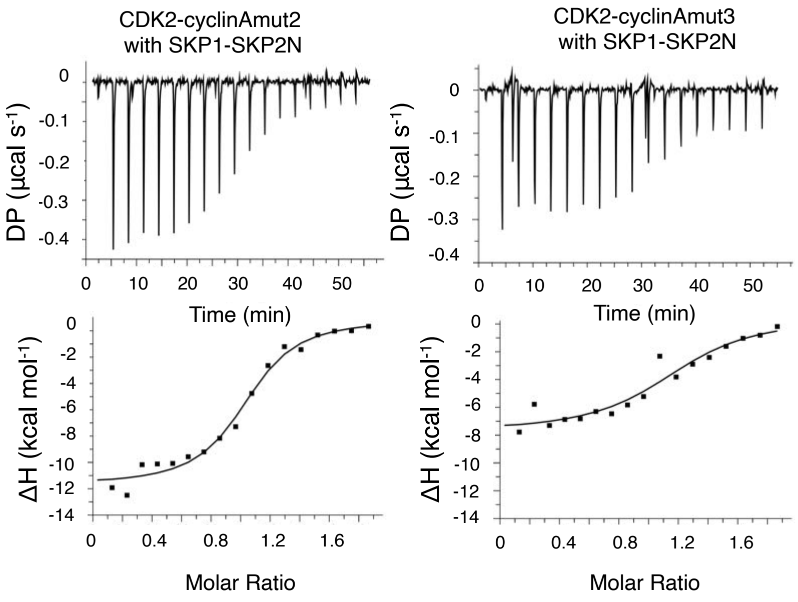
CDK2-cyclinAmut2
with SKP1-SKP2N
CDK2-cyclinAmut3
with SKP1-SKP2N
DP (µcal s-1)
Time (min)
ΔH (kcal mol-1)
Molar Ratio

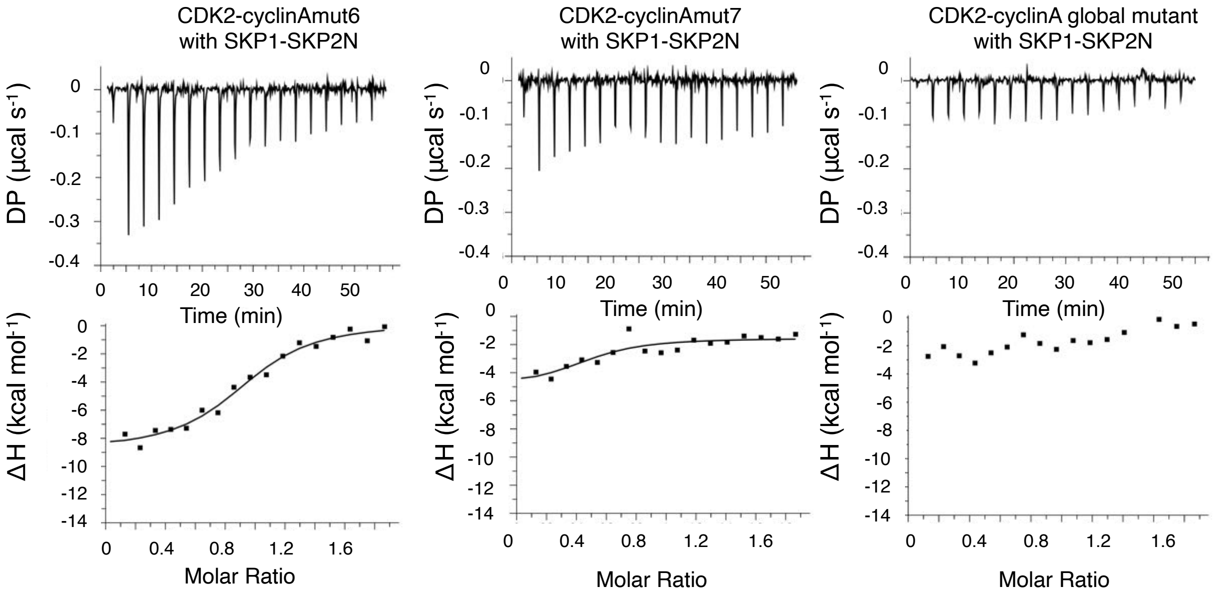

C

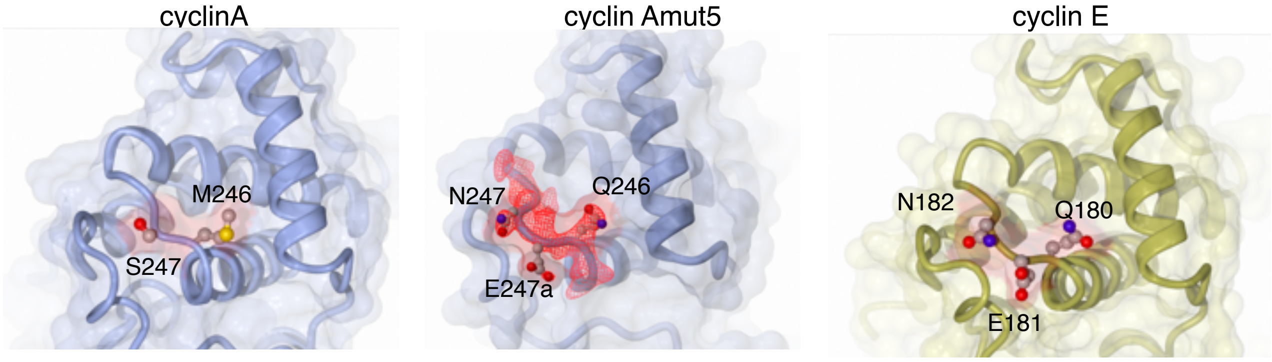

D

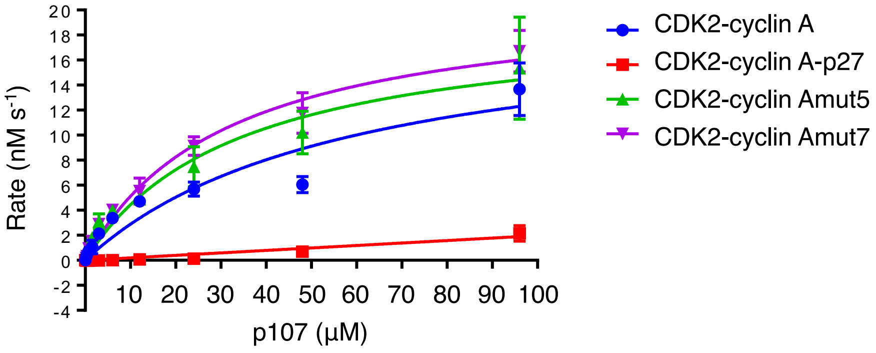

Supplementary Figure S2. Characterization of cyclin A mutants. Related to Table 1. (**A**) Cyclin A mutant locations on the structure of a CDK2-cyclin A-p27 complex (PDB entry 1JSU). The cyclin A surface is rendered in blue, and the CDK2 and p27 folds in green and magenta respectively. Residues mutated in each of the cyclin A mutants are highlighted in

red. (**B**) Isothermal titration calorimetry to measure the interaction between SKP1-SKP2N and mutant CDK2-cyclin A complexes. Thermodynamic parameters and binding constants are tabulated in Table 1. (**C**) Structure of cyclin Amut5. The structure of cyclin Amut5 is shown in the middle panel and compared to that of cyclin A (LHS panel, extracted from PDB entry 1QMZ, [1]) and cyclin E (RHS panel, extracted from PDB entry 1W98 [2]). Cyclin A residues M246 and S247 and cyclin E residues Q180, E181 and N182 (Uniprot entry P24864, numbered as Q165, E166 and N167 in PDB 1W98) are drawn in ball and stick mode. The electron density map that supports the cyclin Amut5 structure at the mutation site is drawn as a red mesh and contoured at 1σ. The location of the SSMS site on the cyclin A structure is also highlighted in red in Figure 1C. This structure adopts an unusual packing in the crystal in which CDK2 N-lobes undergo partial unfolding and domain exchange, but in which the structure of cyclin A is unperturbed. The structures of cyclin A, cyclin E, and cyclin Amut5 overlay closely in the region of the mut5 mutations, although cyclin E and cyclin Amut5 accommodate an insertion where cyclin E residues E181 and N182 replace cyclin A S247. This sequence insertion is one of only two insertions/deletions that distinguish their respective N-terminal cyclin box folds (CBFs). A superposition of cyclin A and cyclin Amut5 indicates that the insertion has not affected the overall cyclin A fold: the extra residue forms a bulge within a surface loop, returning to register at residue 249. As predicted from the cyclin E structure, residue E247a of cyclin Amut5 (analogous to cyclin E E181), orientates into solution and alters the charge of a cleft within cyclin A. (**D**) CDK2-cyclin A and CDK2-cyclin A mutants have comparable catalytic activity. CDK2-cyclin A activity was measured using the ADP-Glo^{TM} assay format towards a peptide substrate derived from the sequence of p107 that contains the sequence SPIK around the site of phosphor-transfer and an RXL recruitment site motif (sequence KRRL) [3]. CDK2-cyclin A-p27(p27 residues 1-106) is not active in this assay (red trace). Kinetic parameters were derived using PRISM (GraphPad) and are presented in Supplementary Table S2. Protein kinase assays were performed in triplicate and error bars correspond to the range of values.

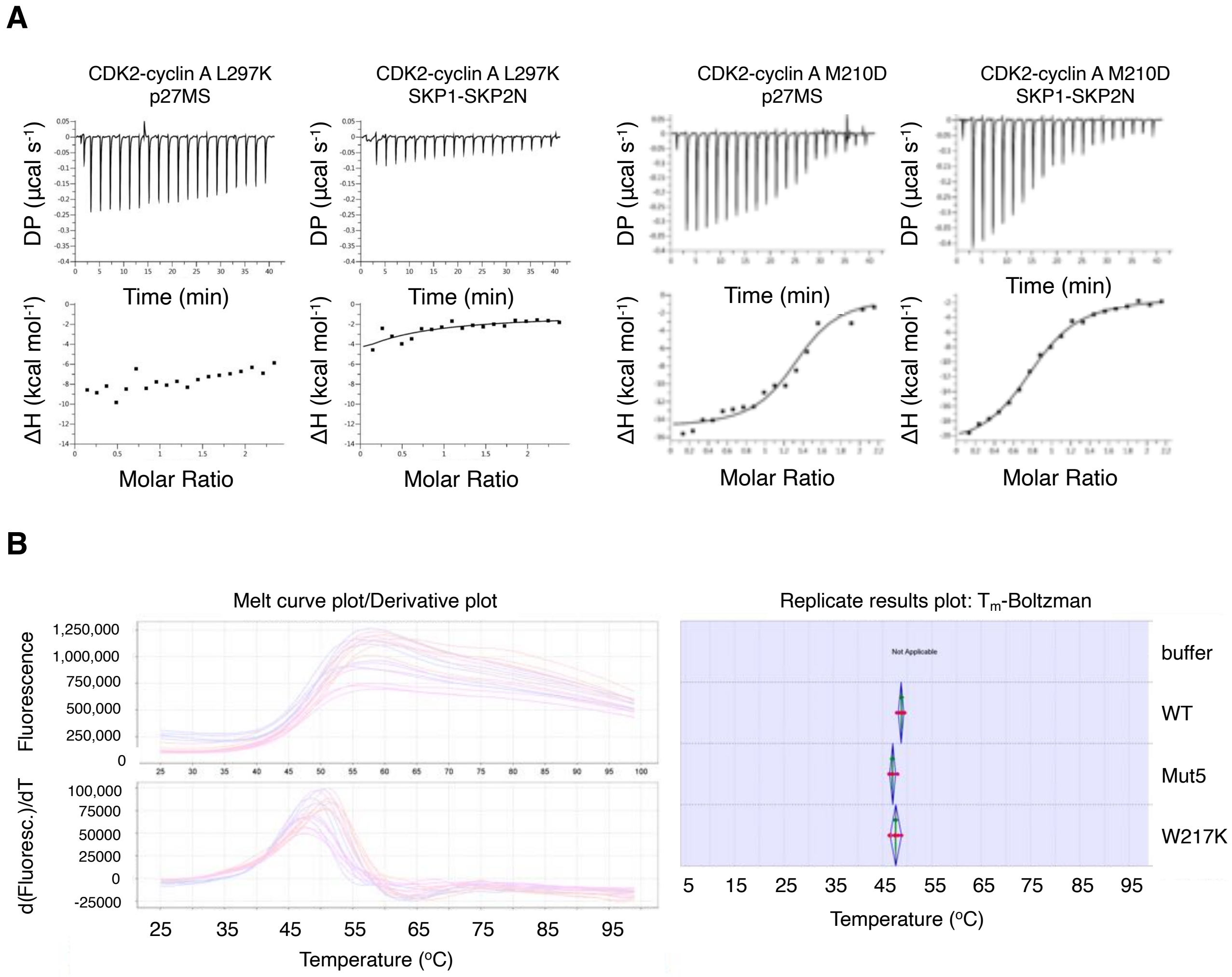

Supplementary Figure S3. Cyclin A mutations distinguish the SKP2 and p27 binding sites. Related to Figure 2.

(**A**) Isothermal titration calorimetry (ITC) to measure the interaction between p27MS (residues 23-51) and SKP1-SKP2N and mutant CDK2-cyclin A complexes. K_d values and reaction conditions are tabulated in Table 1. ITC thermograms are representative of two repeats using independently prepared protein samples. (**B**) Differential scanning fluorimetry to assess the stability of the cyclin A fold. Raw fluorescence data were extracted from the QuantStudio Real-Time PCR software (LHS panel) and analyzed using the Applied Biosystems Protein Thermal Shift software (RHS panel). Derivative T_m values for each complex were extracted and the average values from 3 technical repeats calculated. Red curves, CDK2-cyclin A, magenta curves, CDK2-cyclin Amut5, blue curves CDK2-cyclin AW217K.

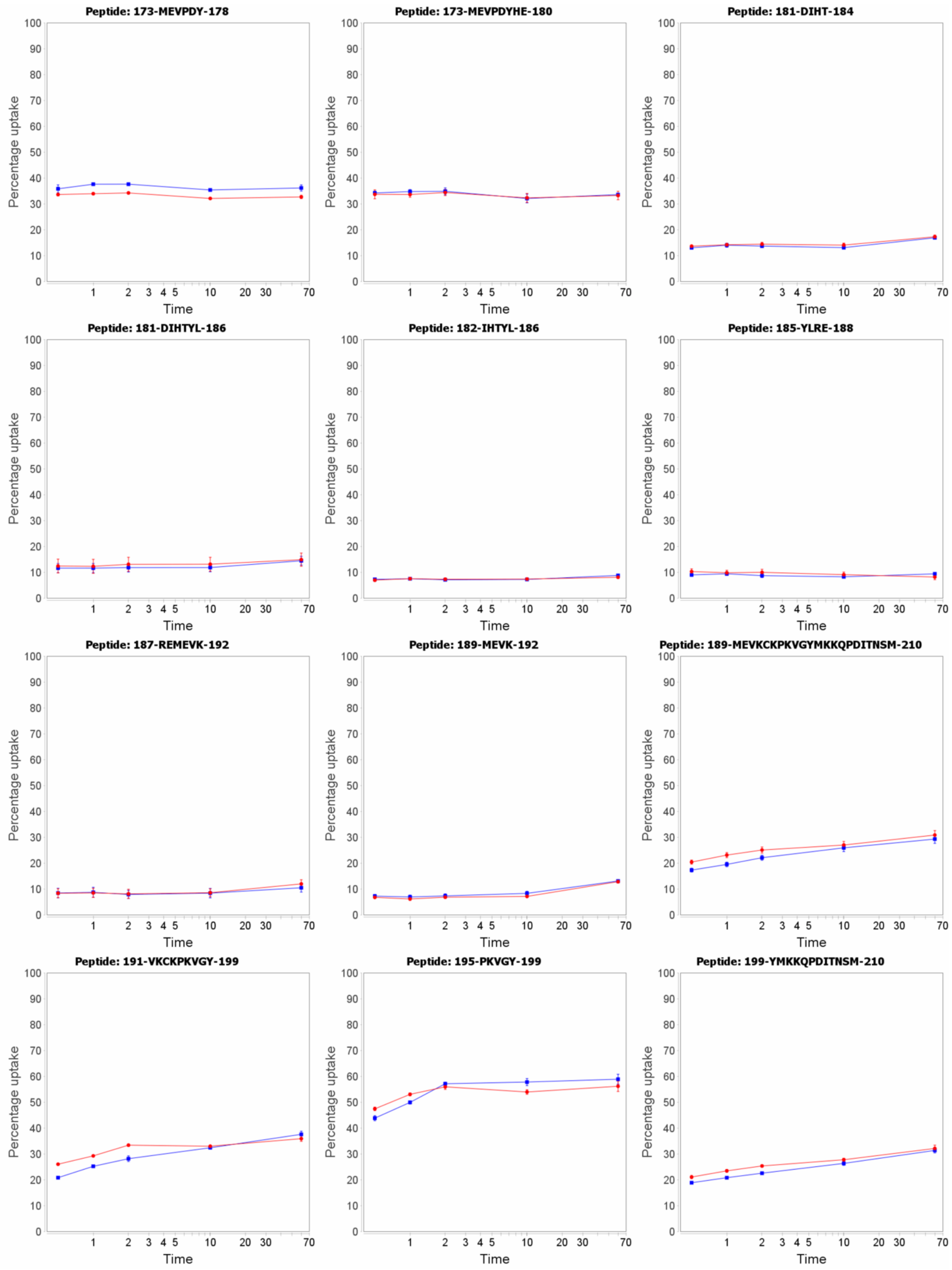
Peptide: 173-MEVPDY-178
Peptide: 173-MEVPDYHE-180
Peptide: 181-DIHT-184
Peptide: 181-DIHTYL-186
Peptide: 182-IHTYL-186
Peptide: 185-YLRE-188
Peptide: 187-REMEVK-192
Peptide: 189-MEVK-192
Peptide: 189-MEVKCKPKVGYMKKQPDITNSM-210
Peptide: 191-VKCKPKVGY-199
Peptide: 195-PKVGY-199
Peptide: 199-YMKKQPDITNSM-210
Percentage uptake
Time

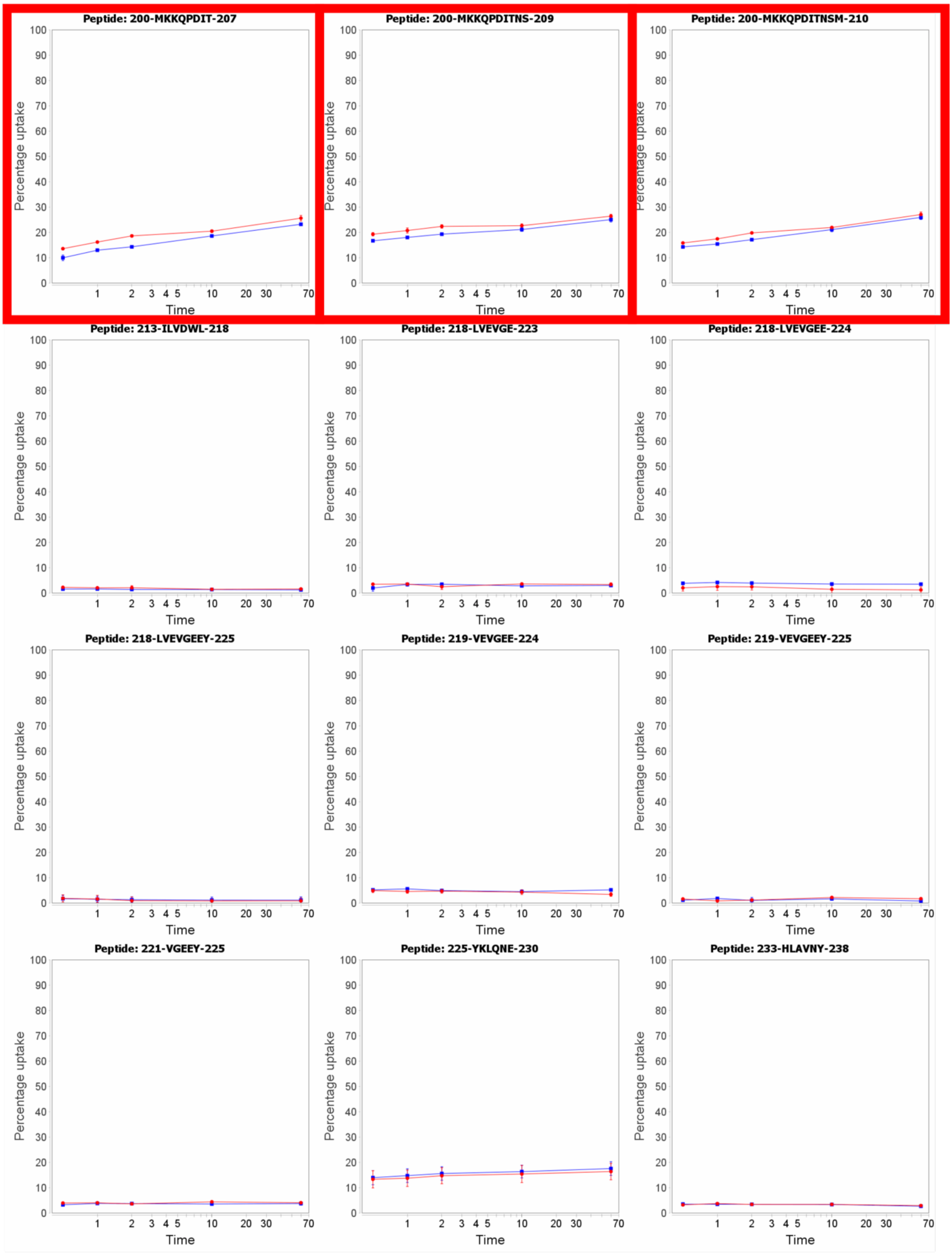
Peptide: 200-MKKQPDIT-207
Peptide: 200-MKKQPDITNS-209
Peptide: 200-MKKQPDITNSM-210
Peptide: 213-ILVDWL-218
Peptide: 218-LVEVGE-223
Peptide: 218-LVEVGEE-224
Peptide: 218-LVEVGEEY-225
Peptide: 219-VEVGEE-224
Peptide: 219-VEVGEEY-225
Peptide: 221-VGEEY-225
Peptide: 225-YKLQNE-230
Peptide: 233-HLAVNY-238
Percentage uptake
Time

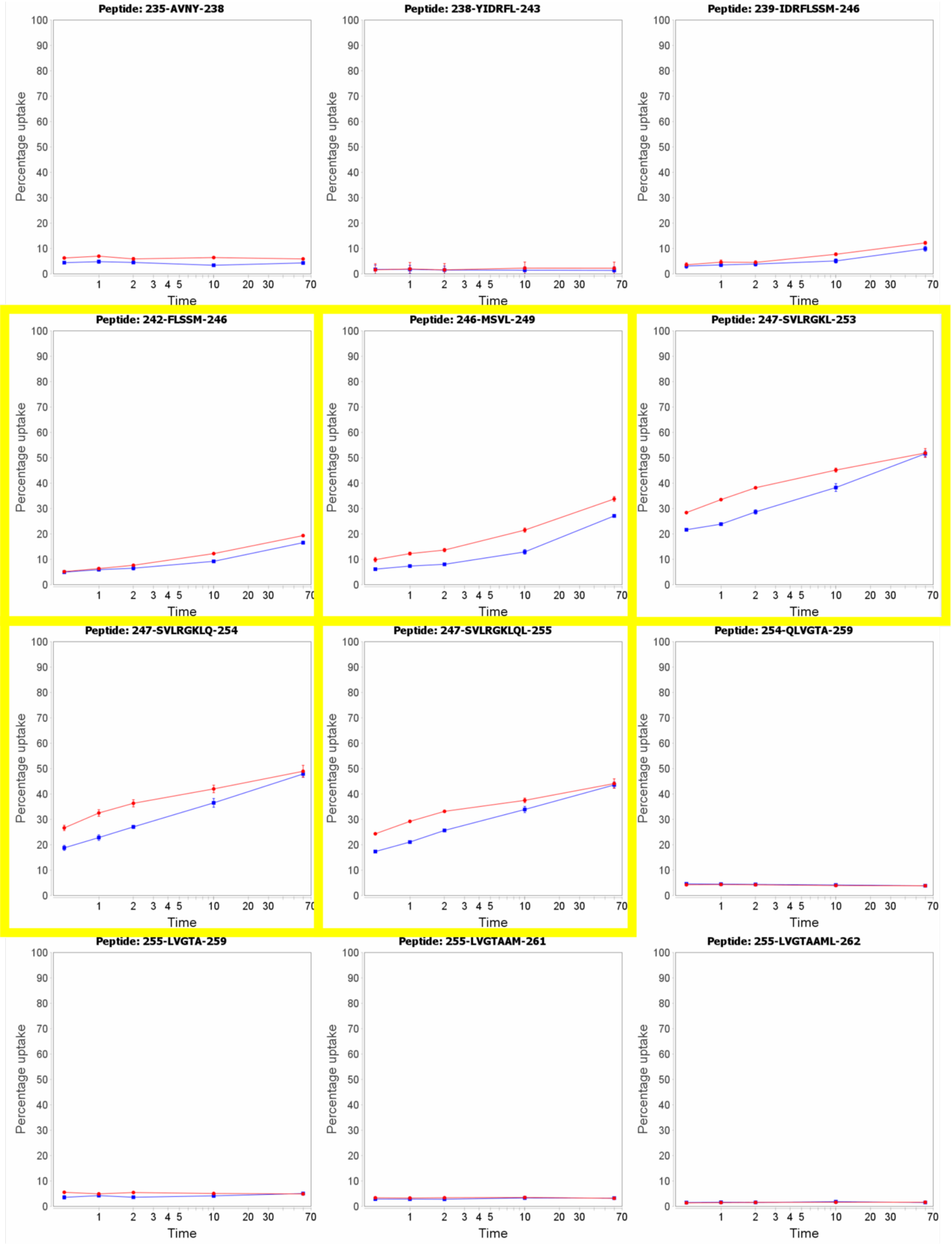
Peptide: 235-AVNY-238
Peptide: 238-YIDRFL-243
Peptide: 239-IDRFLSSM-246
Peptide: 242-FLSSM-246
Peptide: 246-MSVL-249
Peptide: 247-SVLRGKL-253
Peptide: 247-SVLRGKLQ-254
Peptide: 247-SVLRGKLQL-255
Peptide: 254-QLVGTA-259
Peptide: 255-LVGTA-259
Peptide: 255-LVGTAAM-261
Peptide: 255-LVGTAAML-262
Percentage uptake
0 10 20 30 40 50 60 70 80 90 100
Time
1 2 3 4 5 10 20 30 70

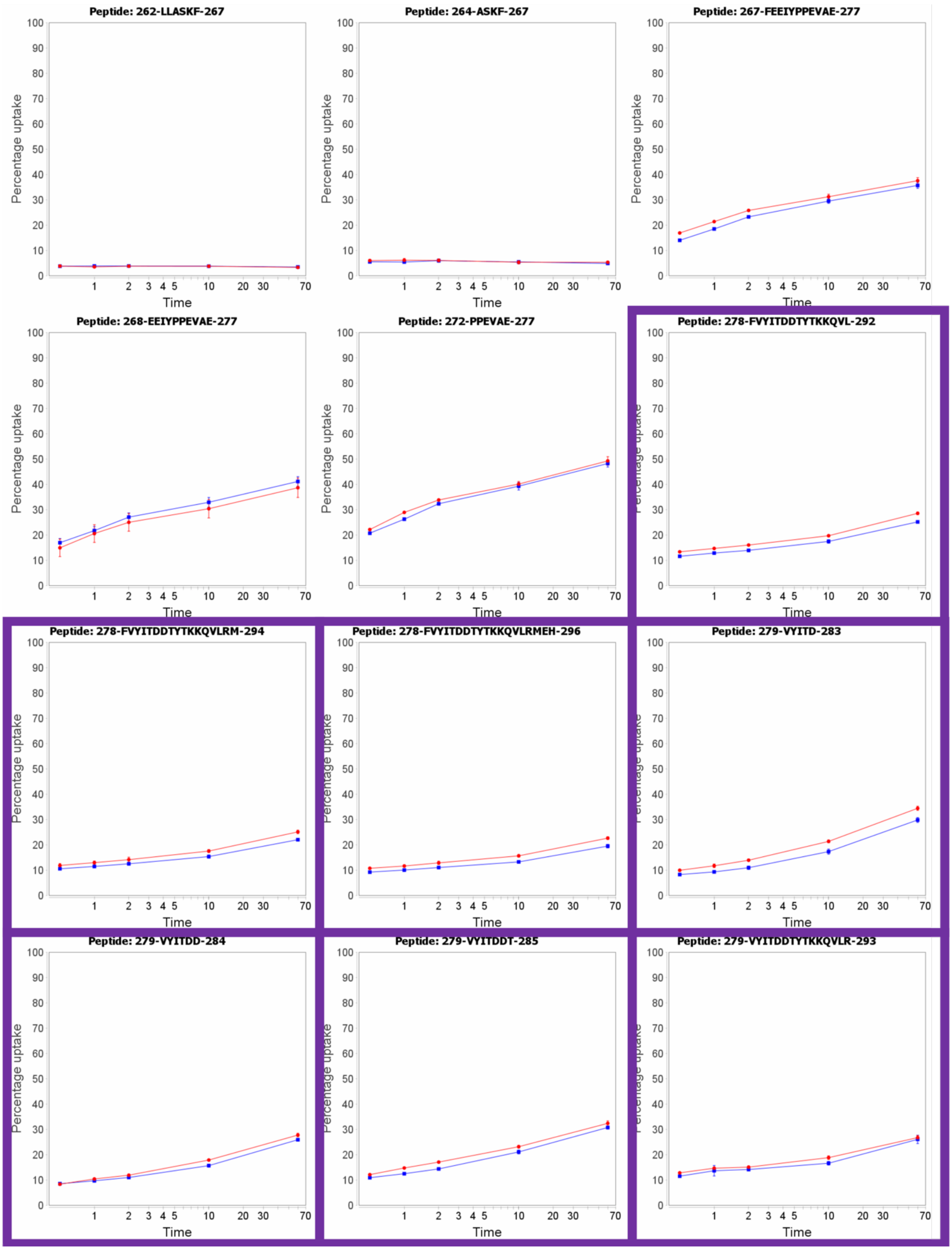
Peptide: 262-LLASKF-267
Peptide: 264-ASKF-267
Peptide: 267-FEEIYPPEVAE-277
Peptide: 268-EEIYPPEVAE-277
Peptide: 272-PPEVAE-277
Peptide: 278-FVYITDDTYTKKQVL-292
Peptide: 278-FVYITDDTYTKKQVLRM-294
Peptide: 278-FVYITDDTYTKKQVLRMEH-296
Peptide: 279-VYITD-283
Peptide: 279-VYITDD-284
Peptide: 279-VYITDDT-285
Peptide: 279-VYITDDTYTKKQVLR-293
Percentage uptake
Time

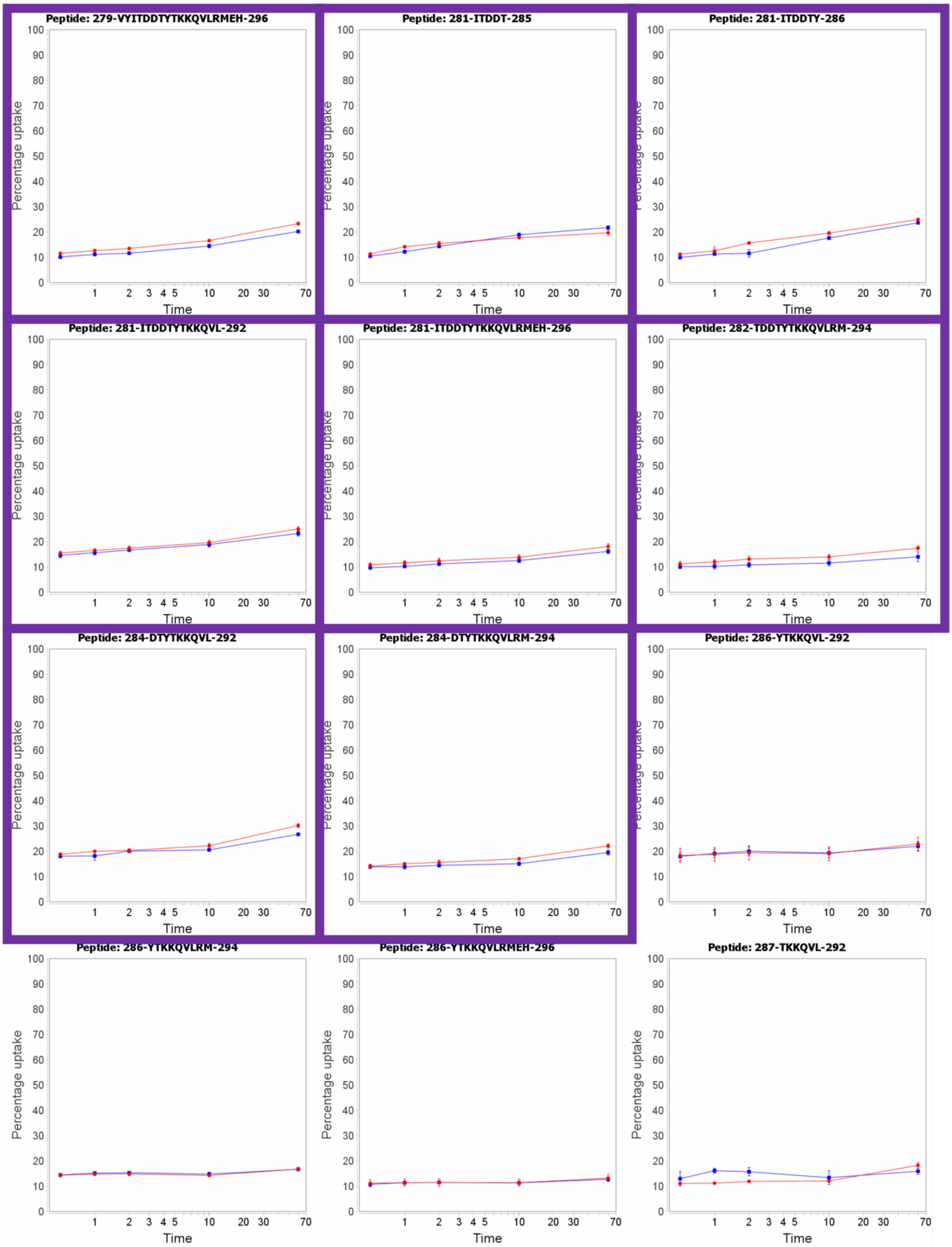
Peptide: 279-VYITDDTYTKKQVLRMEH-296
Peptide: 281-ITDDT-285
Peptide: 281-ITDDTY-286
Peptide: 281-ITDDTYTKKQVL-292
Peptide: 281-ITDDTYTKKQVLRMEH-296
Peptide: 282-TDDTYTKKQVLRM-294
Peptide: 284-DTYTKKQVL-292
Peptide: 284-DTYTKKQVLRM-294
Peptide: 286-YTKKQVL-292
Peptide: 286-YTKKQVLRM-294
Peptide: 286-YTKKQVLRMEH-296
Peptide: 287-TKKQVL-292
Percentage uptake
Time

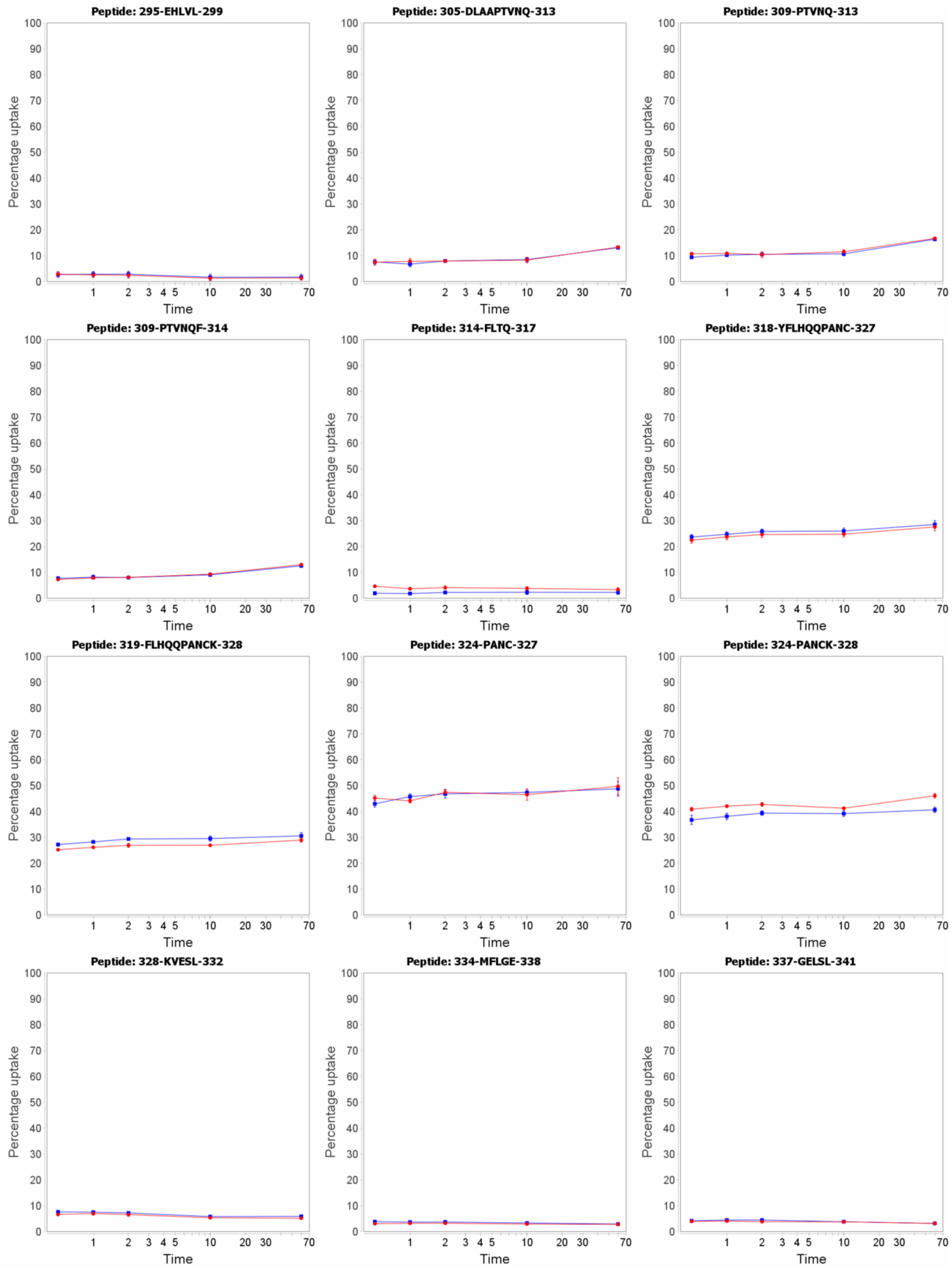
Peptide: 295-EHLVL-299
Peptide: 305-DLAAPTVNQ-313
Peptide: 309-PTVNQ-313
Peptide: 309-PTVNQF-314
Peptide: 314-FLTQ-317
Peptide: 318-YFLHQQPANC-327
Peptide: 319-FLHQQPANCK-328
Peptide: 324-PANC-327
Peptide: 324-PANCK-328
Peptide: 328-KVESL-332
Peptide: 334-MFLGE-338
Peptide: 337-GELSL-341
Percentage uptake
Time
0
10
20
30
40
50
60
70
80
90
100
1
2
3
4
5
10
20
30
70

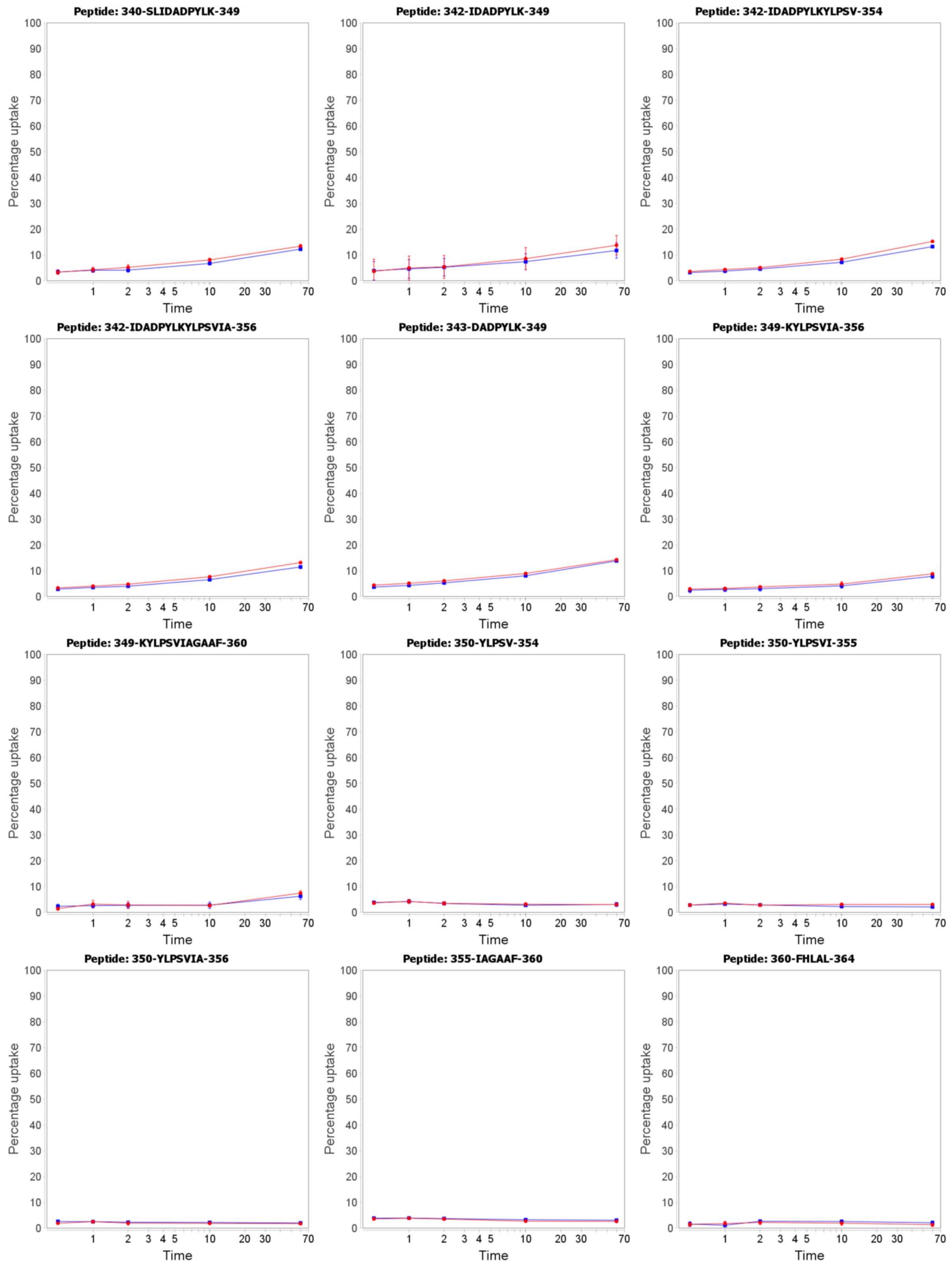

Peptide: 340-SLIDADPYLK-349
Peptide: 342-IDADPYLK-349
Peptide: 342-IDADPYLKYLPSV-354
Peptide: 342-IDADPYLKYLPSVIA-356
Peptide: 343-DADPYLK-349
Peptide: 349-KYLPSVIA-356
Peptide: 349-KYLPSVIAGAAF-360
Peptide: 350-YLPSV-354
Peptide: 350-YLPSVI-355
Peptide: 350-YLPSVIA-356
Peptide: 355-IAGAAF-360
Peptide: 360-FHLAL-364
Percentage uptake
Time
0 10 20 30 40 50 60 70 80 90 100
1 2 3 4 5 10 20 30 70

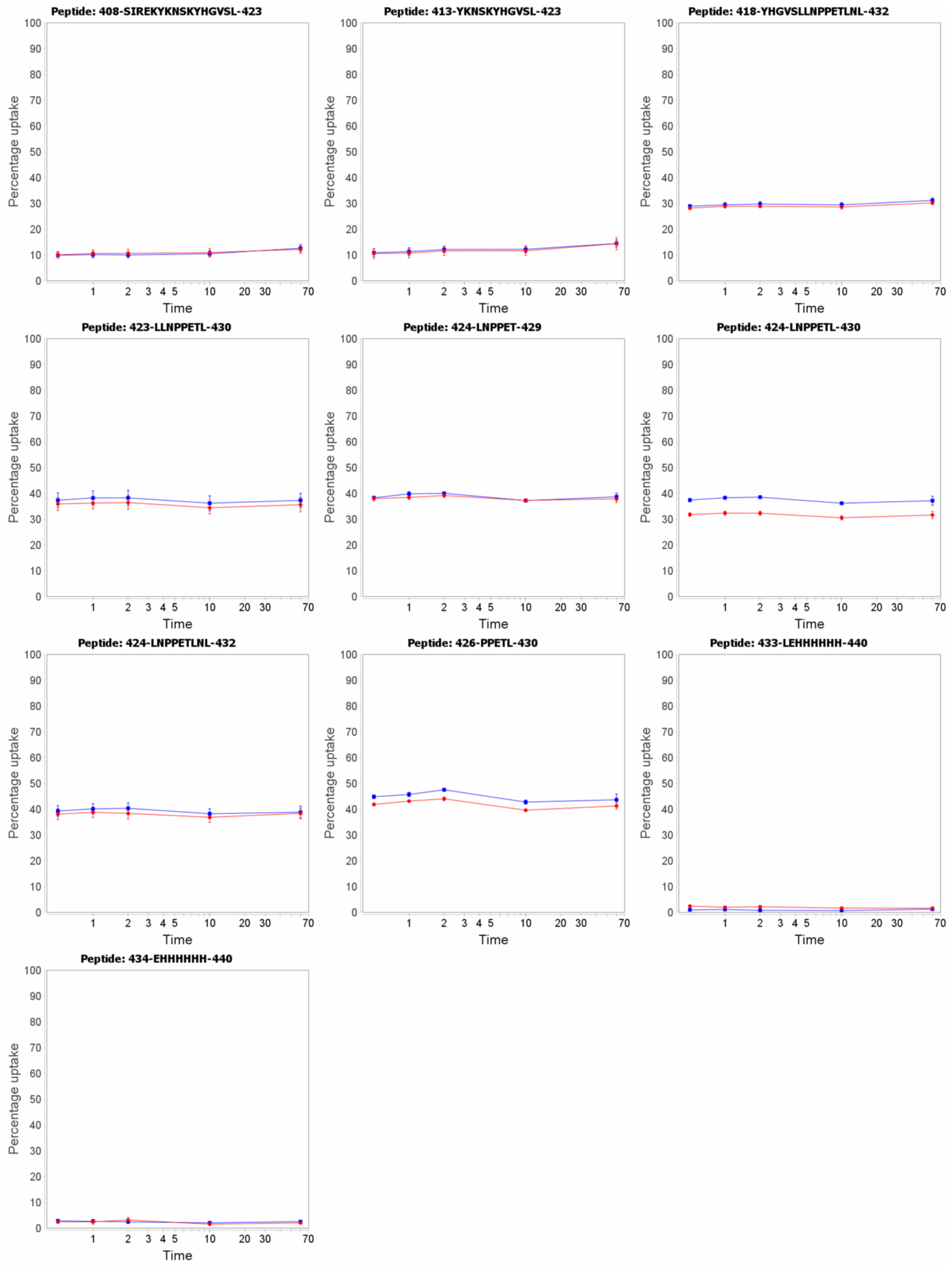
Peptide: 408-SIREKYKNSKYHGVSL-423
Peptide: 413-YKNSKYHGVSL-423
Peptide: 418-YHGVSLLNPPETLNL-432
Peptide: 423-LLNPPETL-430
Peptide: 424-LNPPET-429
Peptide: 424-LNPPETL-430
Peptide: 424-LNPPETLNL-432
Peptide: 426-PPETL-430
Peptide: 433-LEHHHHHH-440
Peptide: 434-EHHHHHH-440
Percentage uptake
Time
0
10
20
30
40
50
60
70
80
90
100
1
2
3
4
5
10
20
30
70

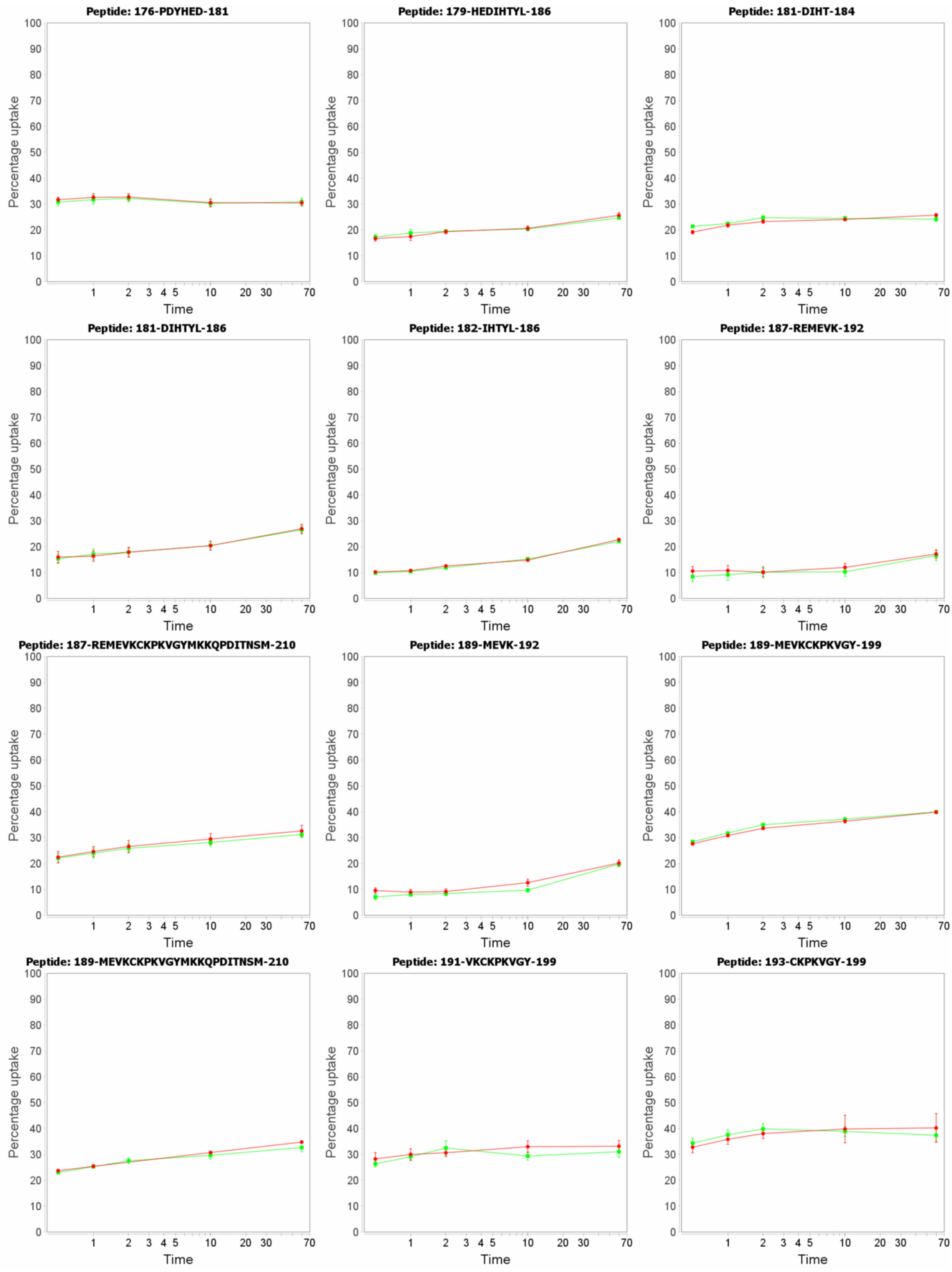
Peptide: 176-PDYHED-181
Peptide: 179-HEDIHTYL-186
Peptide: 181-DIHT-184
Peptide: 181-DIHTYL-186
Peptide: 182-IHTYL-186
Peptide: 187-REMEVK-192
Peptide: 187-REMEVKCKPKVGYMKKQPDITNSM-210
Peptide: 189-MEVK-192
Peptide: 189-MEVKCKPKVGY-199
Peptide: 189-MEVKCKPKVGYMKKQPDITNSM-210
Peptide: 191-VKCKPKVGY-199
Peptide: 193-CKPKVGY-199
Percentage uptake
Time

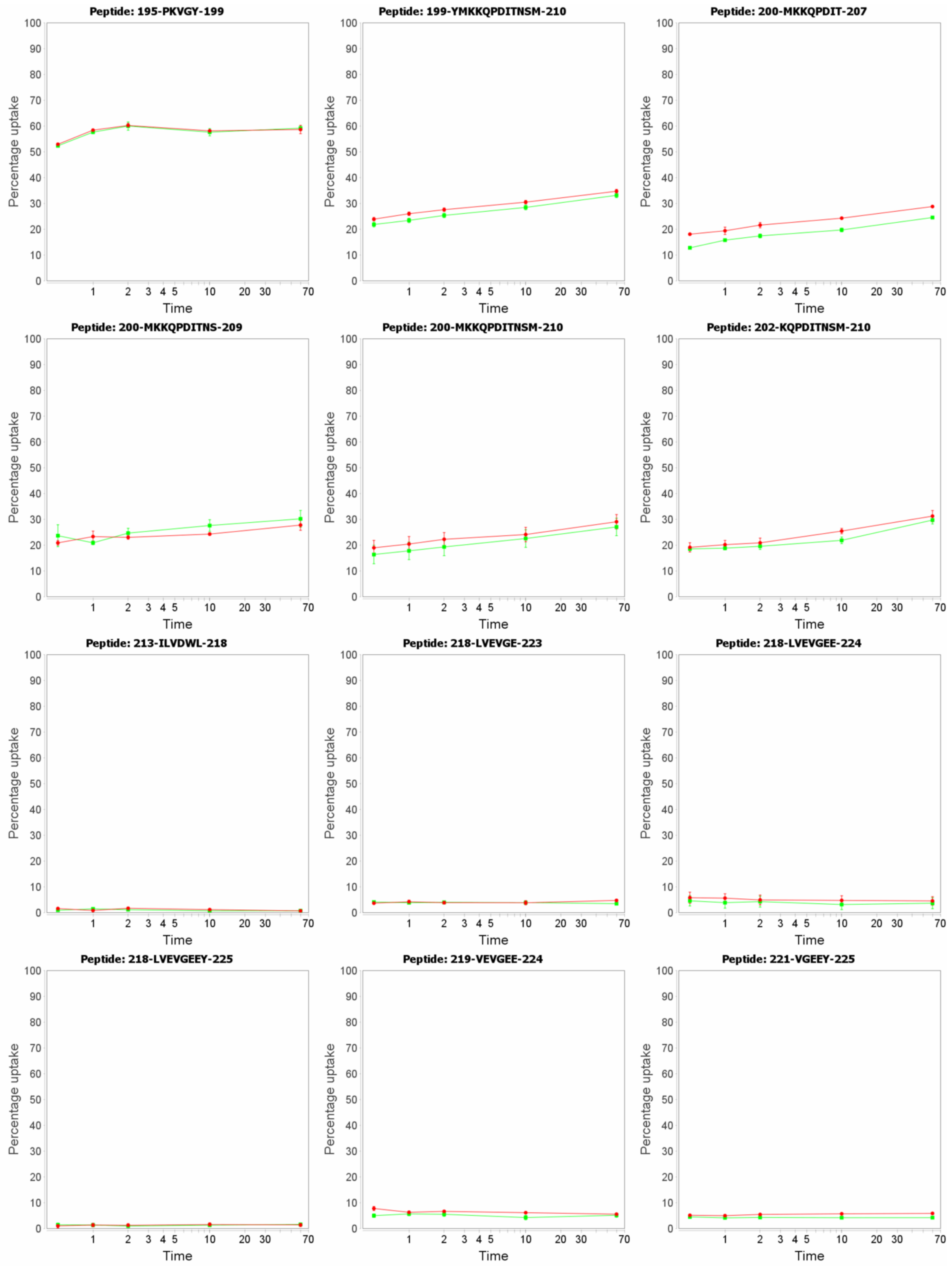
Peptide: 195-PKVGY-199
Peptide: 199-YMKKQPDITNSM-210
Peptide: 200-MKKQPDIT-207
Peptide: 200-MKKQPDITNS-209
Peptide: 200-MKKQPDITNSM-210
Peptide: 202-KQPDITNSM-210
Peptide: 213-ILVDWL-218
Peptide: 218-LVEVGE-223
Peptide: 218-LVEVGEE-224
Peptide: 218-LVEVGEEY-225
Peptide: 219-VEVGEE-224
Peptide: 221-VGEEY-225
Percentage uptake
Time

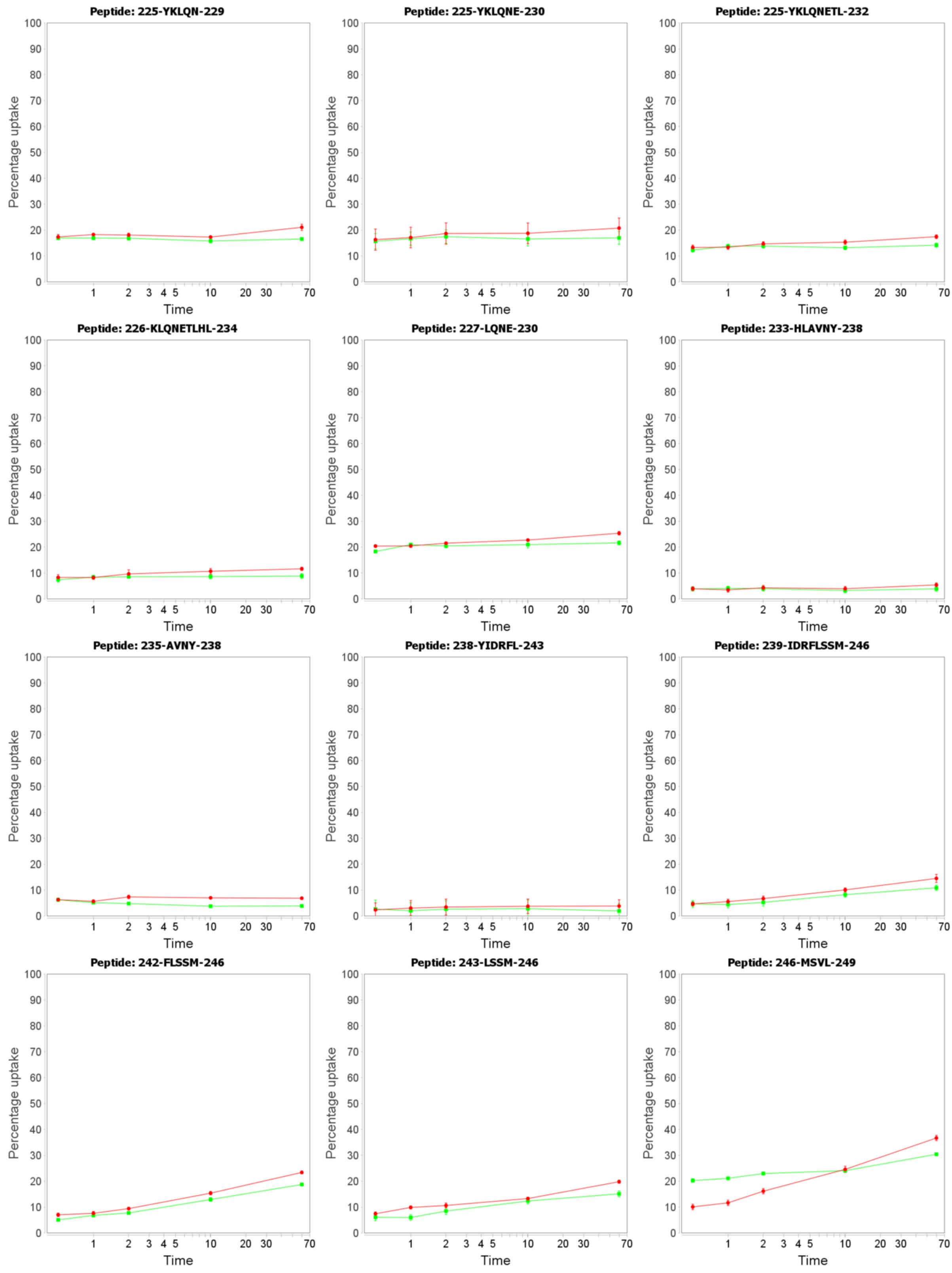
Peptide: 225-YKLQN-229
Peptide: 225-YKLQNE-230
Peptide: 225-YKLQNETL-232
Peptide: 226-KLQNETLHL-234
Peptide: 227-LQNE-230
Peptide: 233-HLAVNY-238
Peptide: 235-AVNY-238
Peptide: 238-YIDRFL-243
Peptide: 239-IDRFLSSM-246
Peptide: 242-FLSSM-246
Peptide: 243-LSSM-246
Peptide: 246-MSVL-249
Percentage uptake
0 10 20 30 40 50 60 70 80 90 100
Time
1 2 3 4 5 10 20 30 70

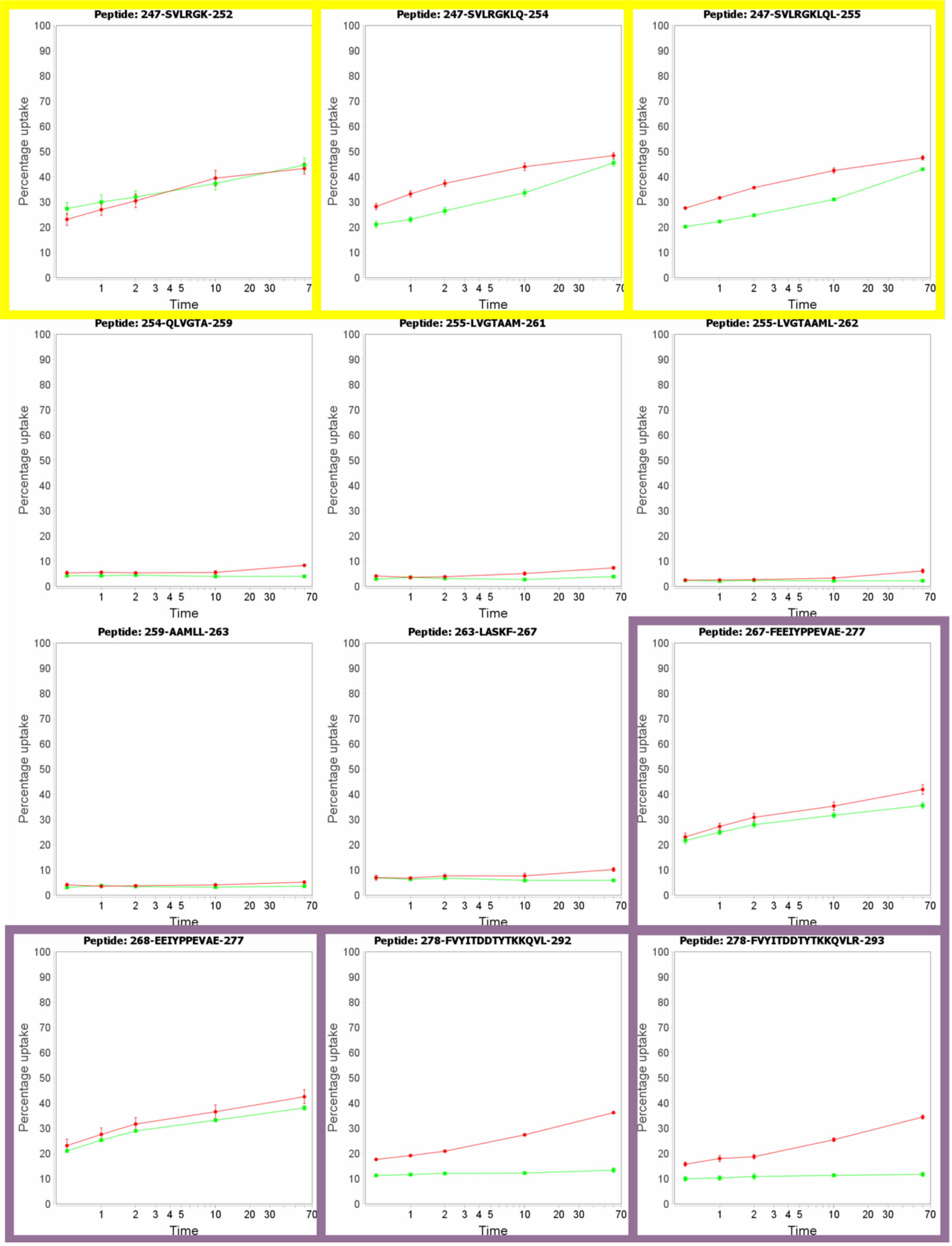
Peptide: 247-SVLRGK-252
Peptide: 247-SVLRGKLQ-254
Peptide: 247-SVLRGKLQL-255
Peptide: 254-QLVGTA-259
Peptide: 255-LVGTAAM-261
Peptide: 255-LVGTAAML-262
Peptide: 259-AAMLL-263
Peptide: 263-LASKF-267
Peptide: 267-FEEIYPPEVAE-277
Peptide: 268-EEIYPPEVAE-277
Peptide: 278-FVYITDDTYTKKQVL-292
Peptide: 278-FVYITDDTYTKKQVLR-293
Percentage uptake
Time

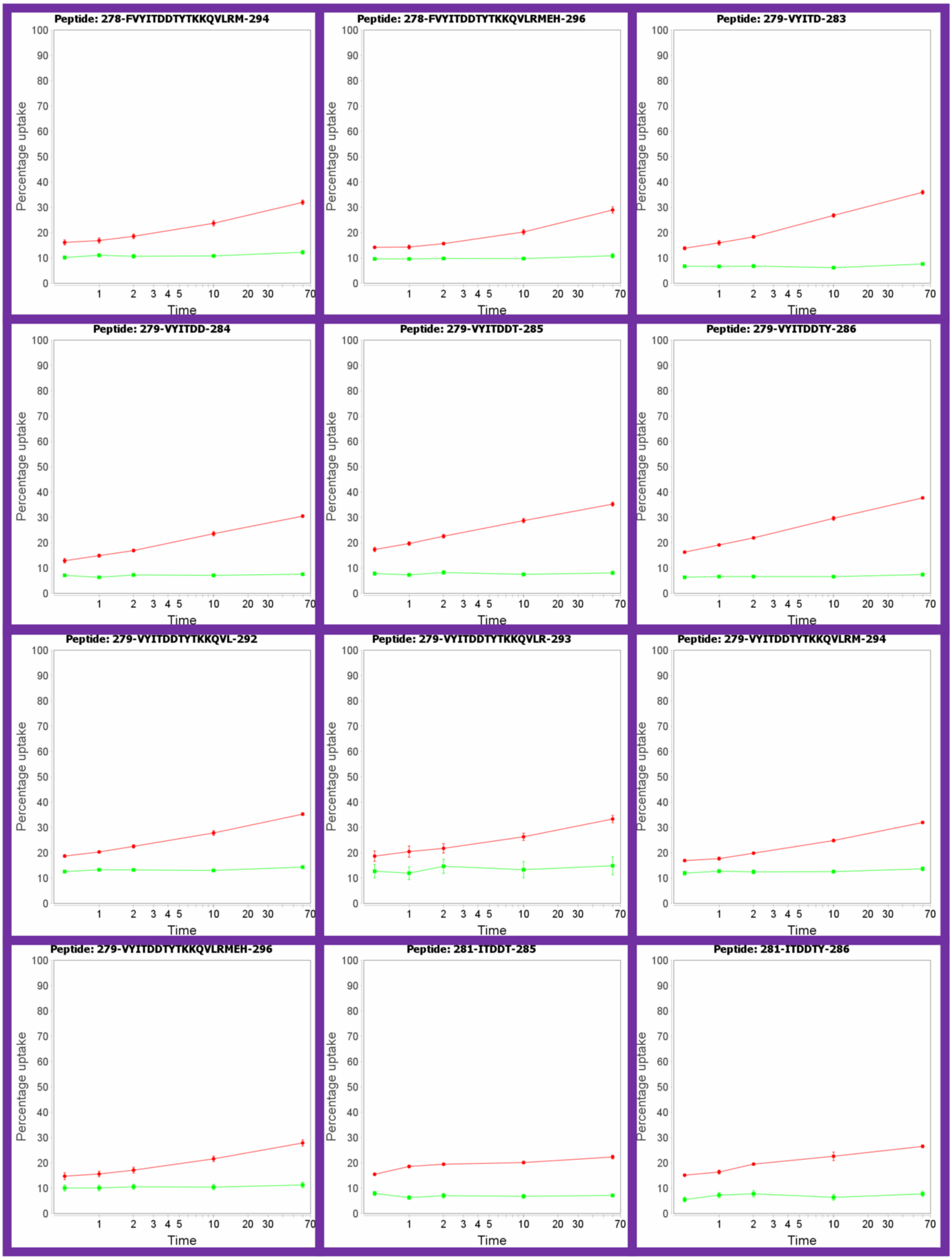
Peptide: 278-FVYITDDTYTKKQVLRM-294
Peptide: 278-FVYITDDTYTKKQVLRMEH-296
Peptide: 279-VYITD-283
Peptide: 279-VYITDD-284
Peptide: 279-VYITDDT-285
Peptide: 279-VYITDDTY-286
Peptide: 279-VYITDDTYTKKQVL-292
Peptide: 279-VYITDDTYTKKQVLR-293
Peptide: 279-VYITDDTYTKKQVLRM-294
Peptide: 279-VYITDDTYTKKQVLRMEH-296
Peptide: 281-ITDDT-285
Peptide: 281-ITDDTY-286
Percentage uptake
Time

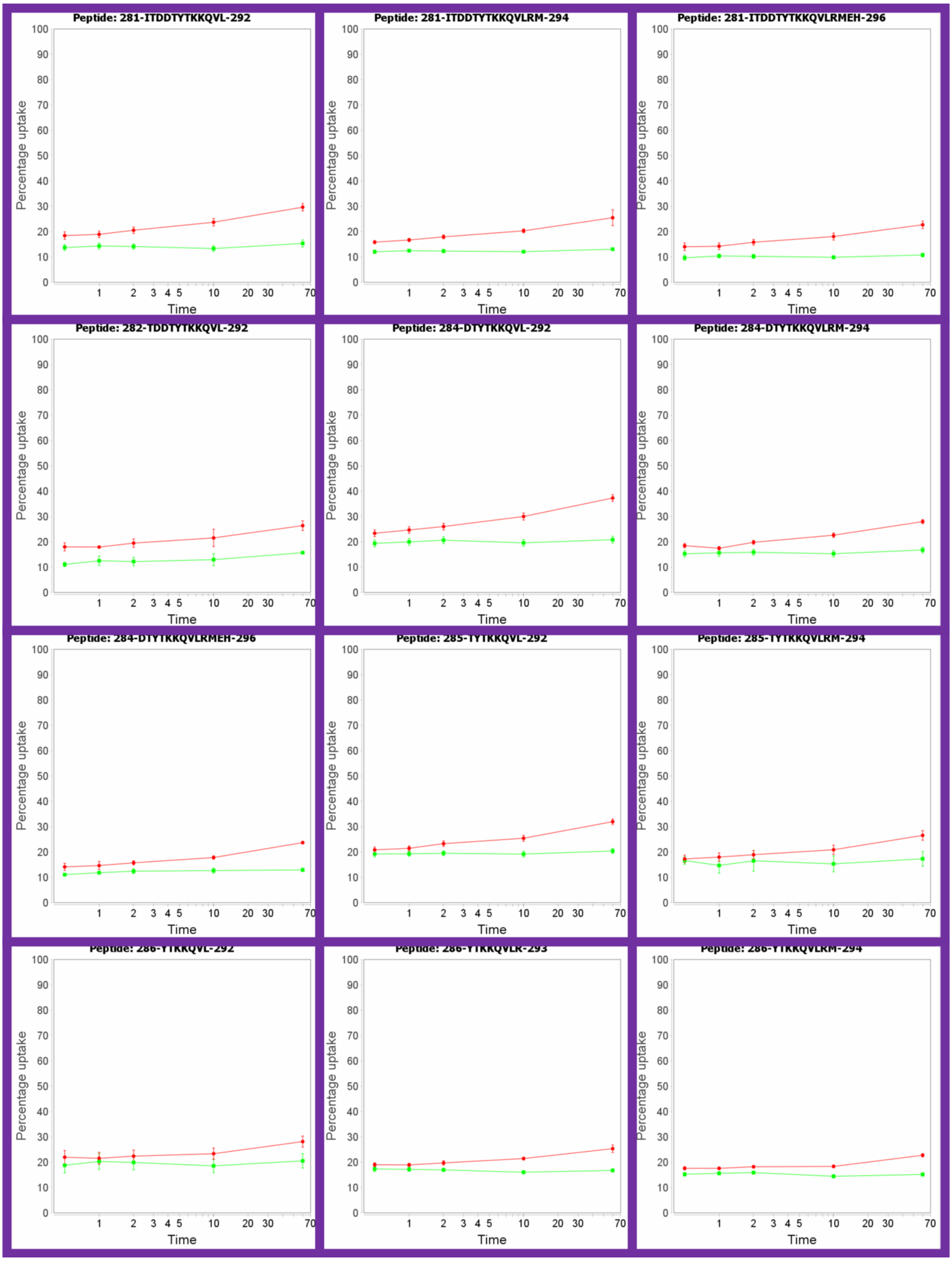
Peptide: 281-ITDDTYTKKQVL-292
Peptide: 281-ITDDTYTKKQVLRM-294
Peptide: 281-ITDDTYTKKQVLRMEH-296
Peptide: 282-TDDTYTKKQVL-292
Peptide: 284-DTYTKKQVL-292
Peptide: 284-DTYTKKQVLRM-294
Peptide: 284-DTYTKKQVLRMEH-296
Peptide: 285-TYTKKQVL-292
Peptide: 285-TYTKKQVLRM-294
Peptide: 286-YTKKQVL-292
Peptide: 286-YTKKQVLR-293
Peptide: 286-YTKKQVLRM-294
Percentage uptake
Time

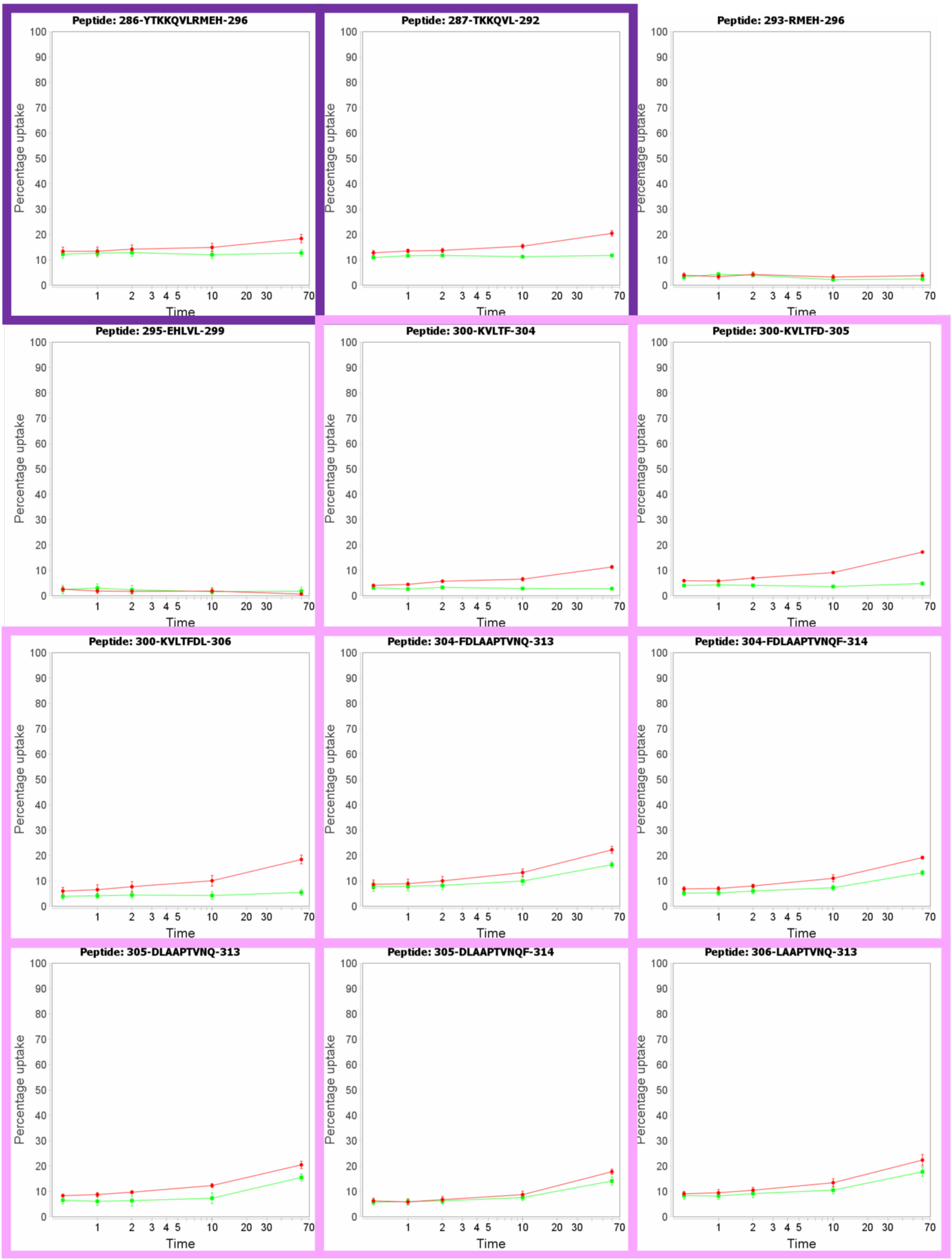
Peptide: 286-YTKKQVLRMEH-296
Peptide: 287-TKKQVL-292
Peptide: 293-RMEH-296
Peptide: 295-EHLVL-299
Peptide: 300-KVLTF-304
Peptide: 300-KVLTFD-305
Peptide: 300-KVLTFDL-306
Peptide: 304-FDLAAPTVNQ-313
Peptide: 304-FDLAAPTVNQF-314
Peptide: 305-DLAAPTVNQ-313
Peptide: 305-DLAAPTVNQF-314
Peptide: 306-LAAPTVNQ-313
Percentage uptake
Time
0
10
20
30
40
50
60
70
80
90
100
1
2
3
4
5
10
20
30
70

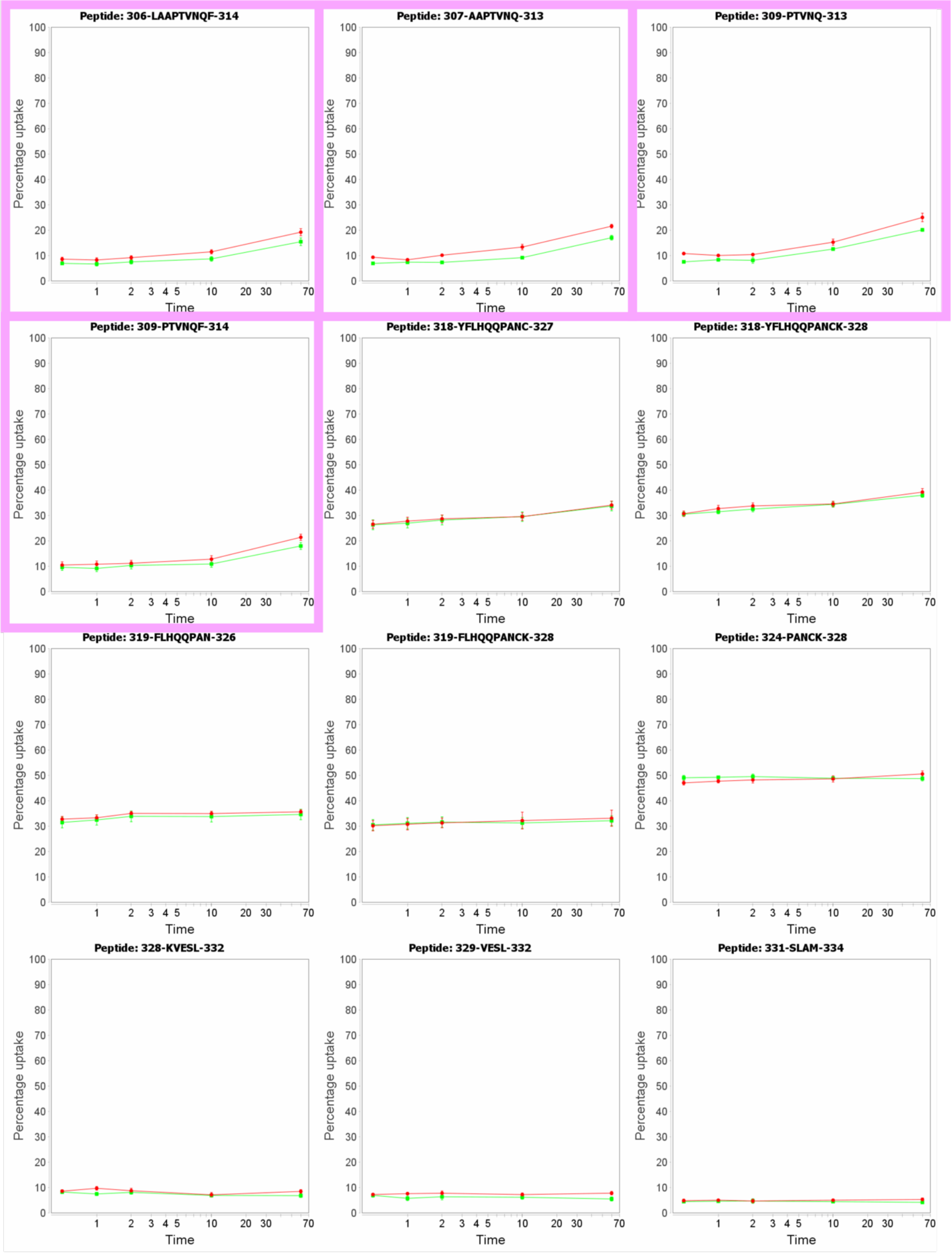
Peptide: 306-LAAPTVNQF-314
Peptide: 307-AAPTVNQ-313
Peptide: 309-PTVNQ-313
Peptide: 309-PTVNQF-314
Peptide: 318-YFLHQQPANC-327
Peptide: 318-YFLHQQPANCK-328
Peptide: 319-FLHQQPAN-326
Peptide: 319-FLHQQPANCK-328
Peptide: 324-PANCK-328
Peptide: 328-KVESL-332
Peptide: 329-VESL-332
Peptide: 331-SLAM-334
Percentage uptake
Time
0
10
20
30
40
50
60
70
80
90
100
1
2
3
4
5
10
20
30
70

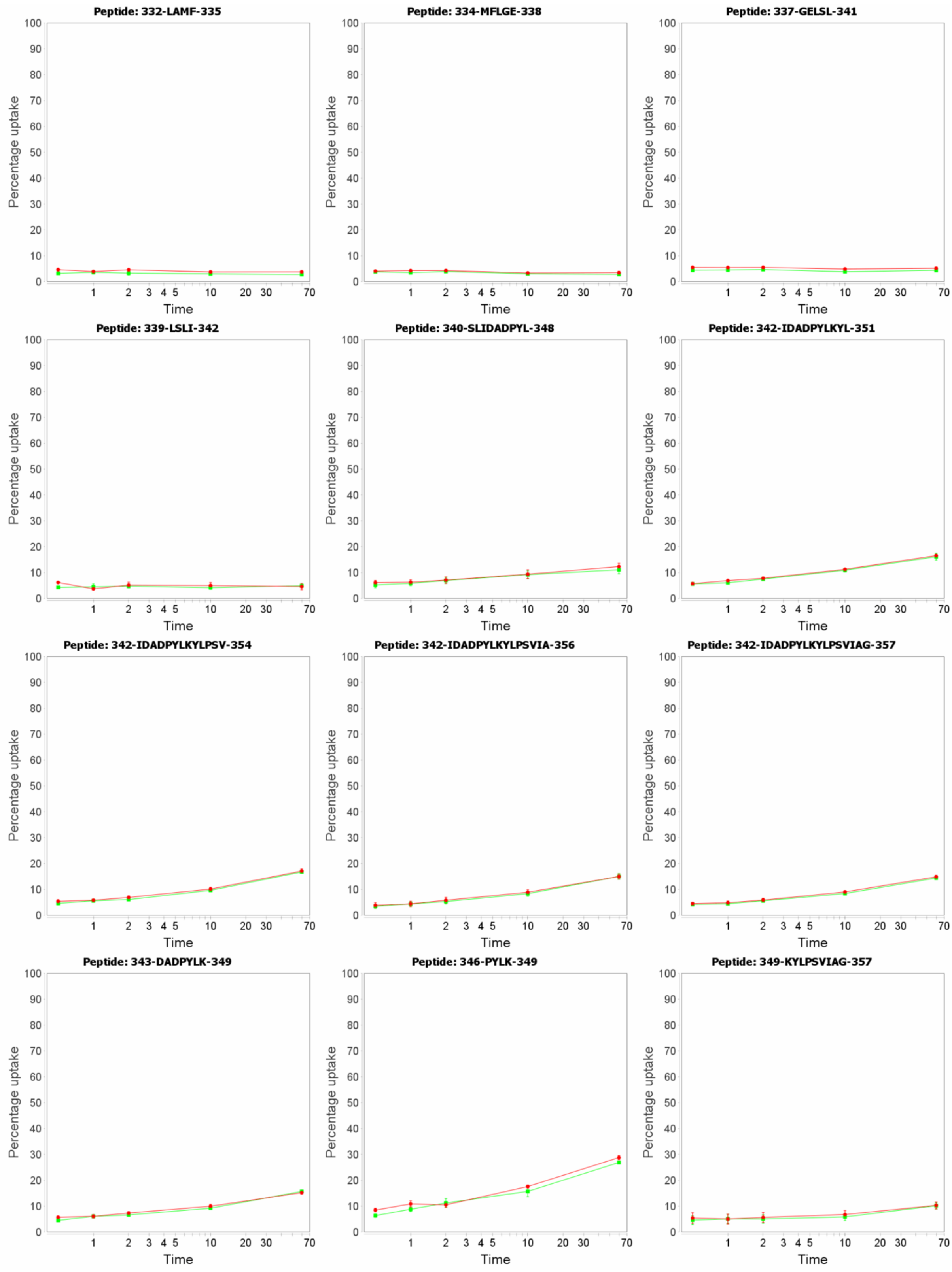
Peptide: 332-LAMF-335
Peptide: 334-MFLGE-338
Peptide: 337-GELSL-341
Peptide: 339-LSLI-342
Peptide: 340-SLIDADPYL-348
Peptide: 342-IDADPYLKYL-351
Peptide: 342-IDADPYLKYLPSV-354
Peptide: 342-IDADPYLKYLPSVIA-356
Peptide: 342-IDADPYLKYLPSVIAG-357
Peptide: 343-DADPYLK-349
Peptide: 346-PYLK-349
Peptide: 349-KYLPSVIAG-357
Percentage uptake
0 10 20 30 40 50 60 70 80 90 100
Time
1 2 3 4 5 10 20 30 70

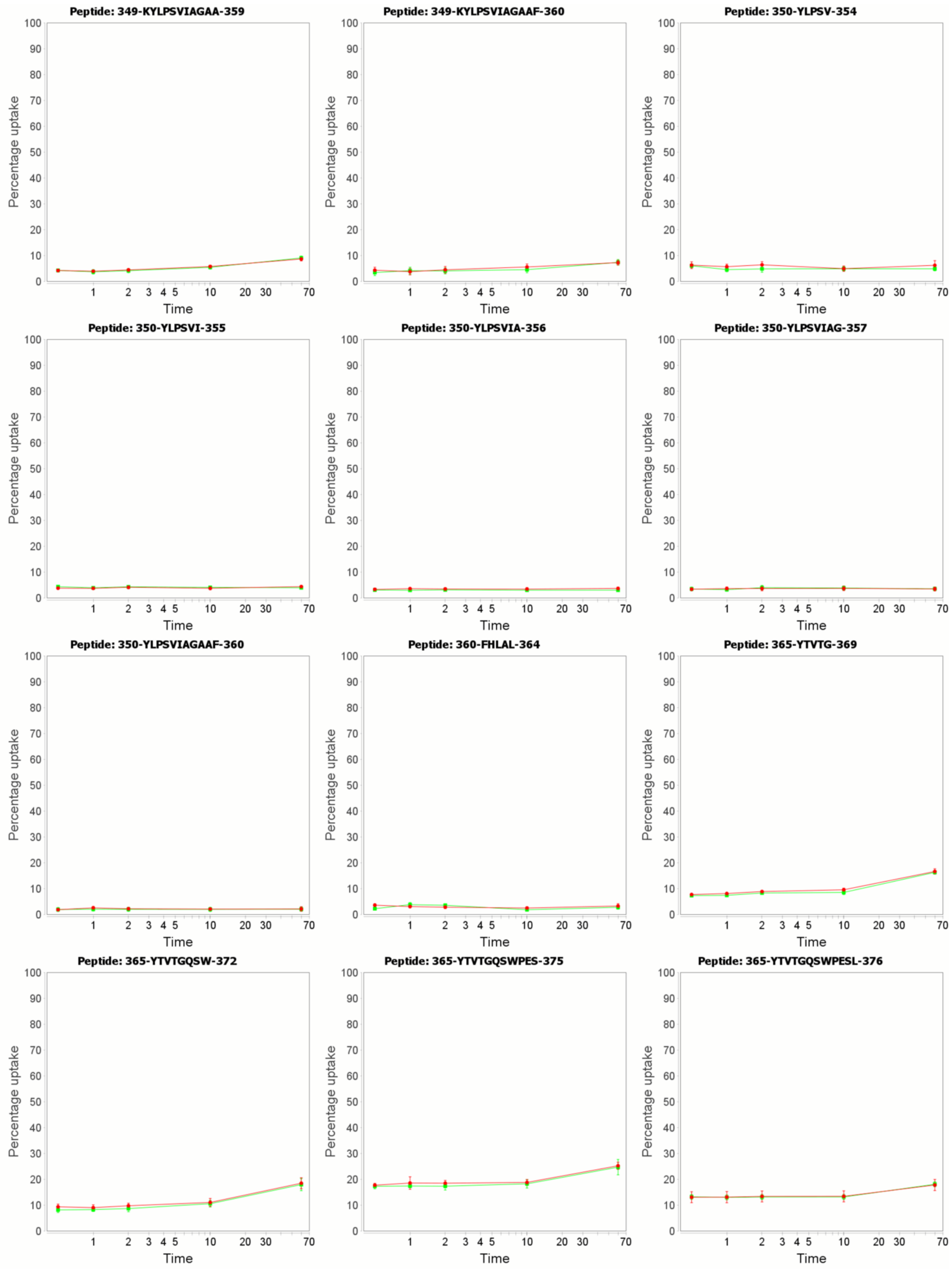
Peptide: 349-KYLPSVIAGAA-359
Peptide: 349-KYLPSVIAGAAF-360
Peptide: 350-YLPSV-354
Peptide: 350-YLPSVI-355
Peptide: 350-YLPSVIA-356
Peptide: 350-YLPSVIAG-357
Peptide: 350-YLPSVIAGAAF-360
Peptide: 360-FHLAL-364
Peptide: 365-YTVTG-369
Peptide: 365-YTVTGQSW-372
Peptide: 365-YTVTGQSWPES-375
Peptide: 365-YTVTGQSWPESL-376
Percentage uptake
0 10 20 30 40 50 60 70 80 90 100
Time
1 2 3 4 5 10 20 30 70

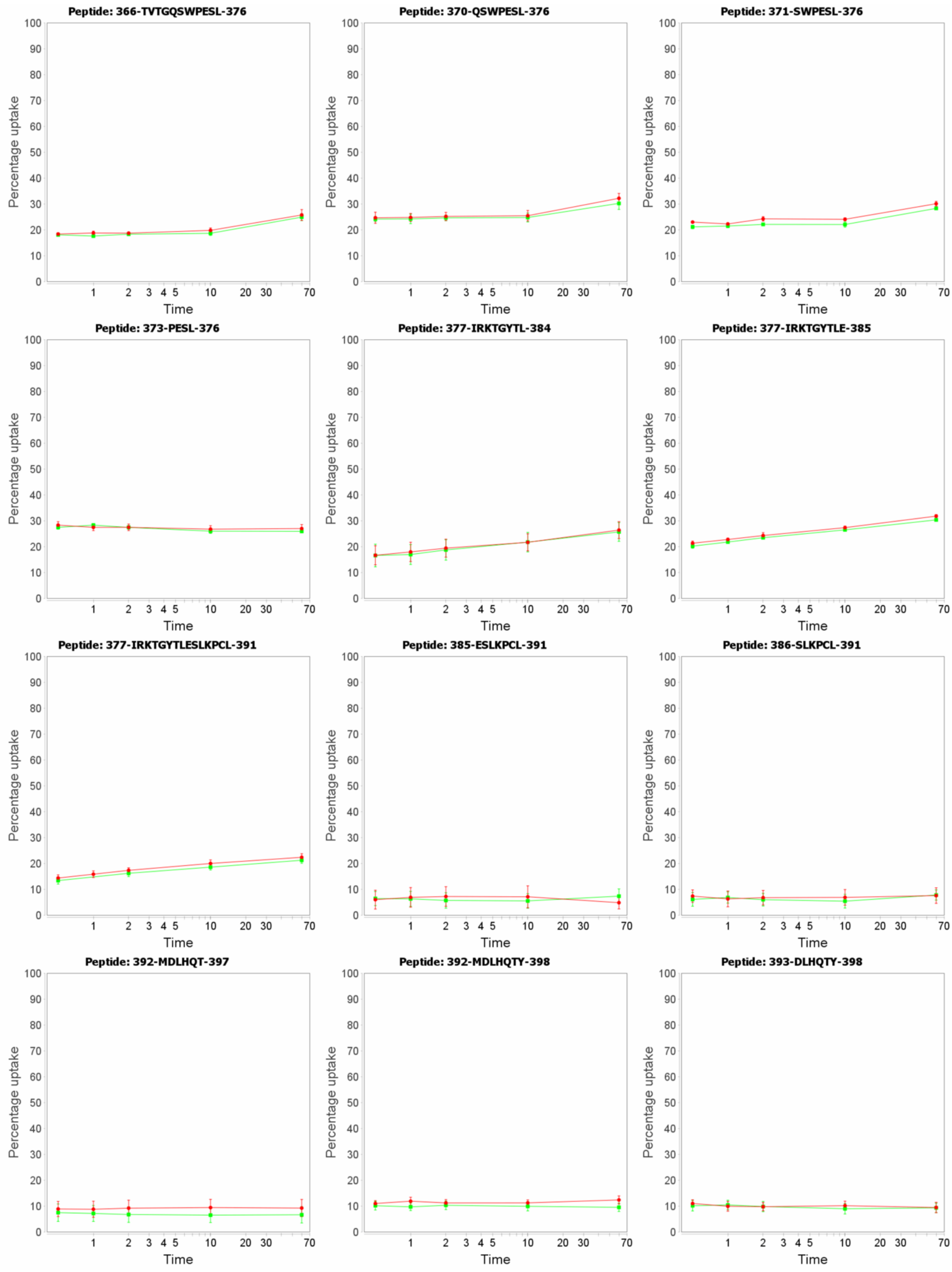

Peptide: 366-TVTGQSWPESL-376
Peptide: 370-QSWPESL-376
Peptide: 371-SWPESL-376
Peptide: 373-PESL-376
Peptide: 377-IRKTGYTL-384
Peptide: 377-IRKTGYTLE-385
Peptide: 377-IRKTGYTLESLKPCL-391
Peptide: 385-ESLKPCL-391
Peptide: 386-SLKPCL-391
Peptide: 392-MDLHQT-397
Peptide: 392-MDLHQTY-398
Peptide: 393-DLHQTY-398
Percentage uptake
0 10 20 30 40 50 60 70 80 90 100
Time
1 2 3 4 5 10 20 30 70

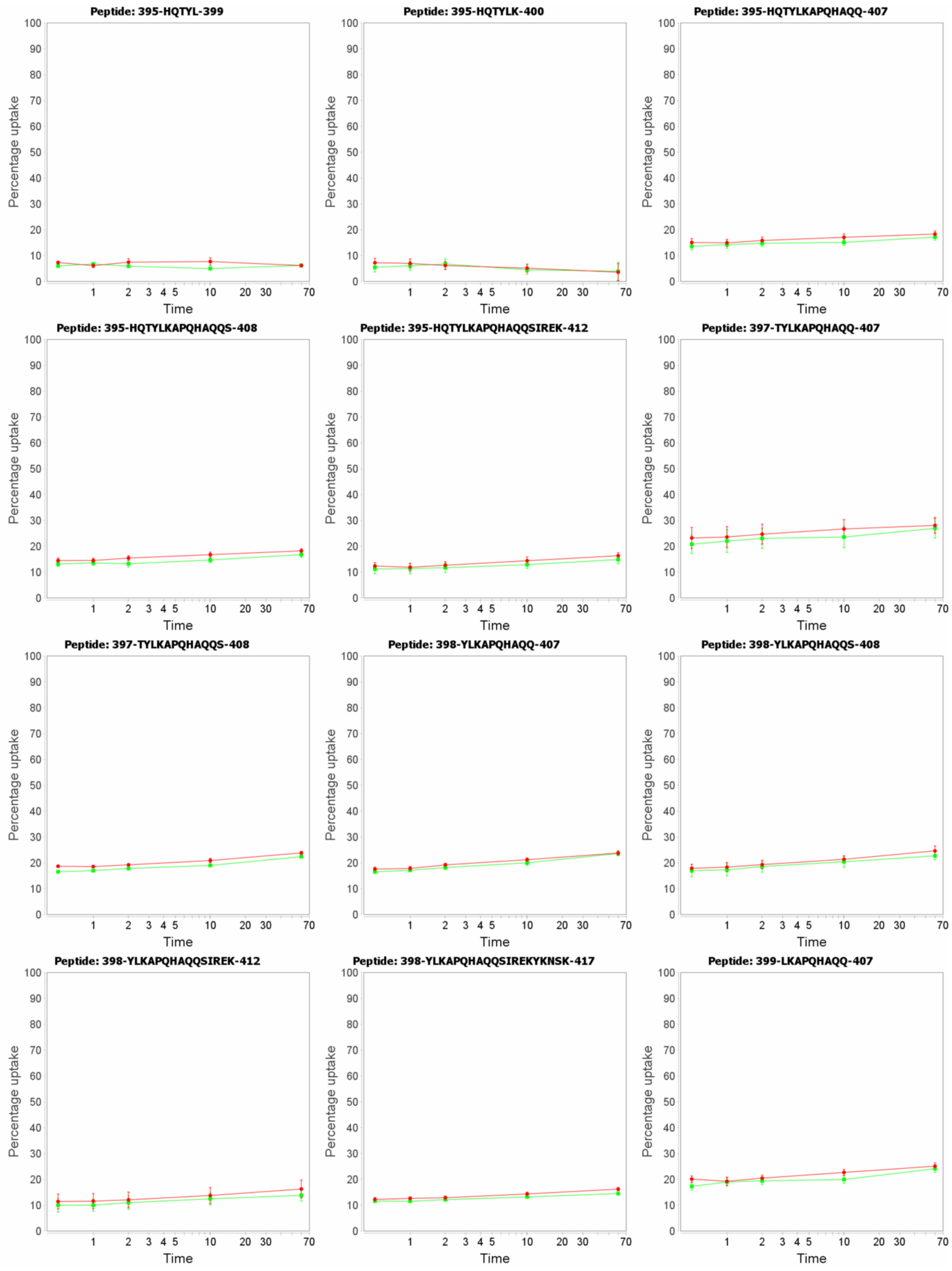
Peptide: 395-HQTYL-399
Peptide: 395-HQTYLK-400
Peptide: 395-HQTYLKAPQHAQQ-407
Peptide: 395-HQTYLKAPQHAQQS-408
Peptide: 395-HQTYLKAPQHAQQSIREK-412
Peptide: 397-TYLKAPQHAQQ-407
Peptide: 397-TYLKAPQHAQQS-408
Peptide: 398-YLKAPQHAQQ-407
Peptide: 398-YLKAPQHAQQS-408
Peptide: 398-YLKAPQHAQQSIREK-412
Peptide: 398-YLKAPQHAQQSIREKYKNSK-417
Peptide: 399-LKAPQHAQQ-407
Percentage uptake
0
10
20
30
40
50
60
70
80
90
100
Time
1
2
3
4
5
10
20
30
70

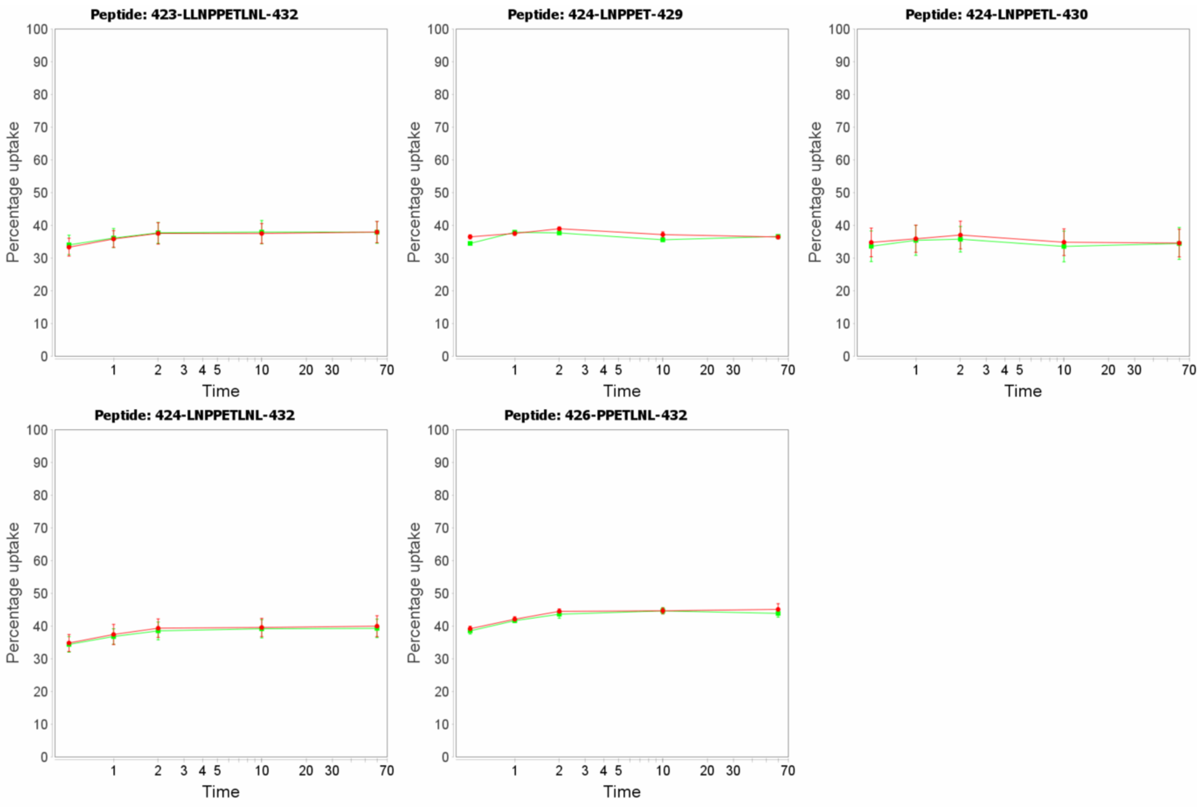

Supplementary Figure S4. Hydrogen-deuterium exchange mass spectrometry to identify the cyclin A SKP2 binding site. Related to Figure 3. Uptake plots for cyclin A peptides in the absence (red) or presence of SKP1-Δ20SKP2N (blue) or p27M (red, green). Time in minutes. Highlighted peptides are boxed, coloring as in Figure 3.

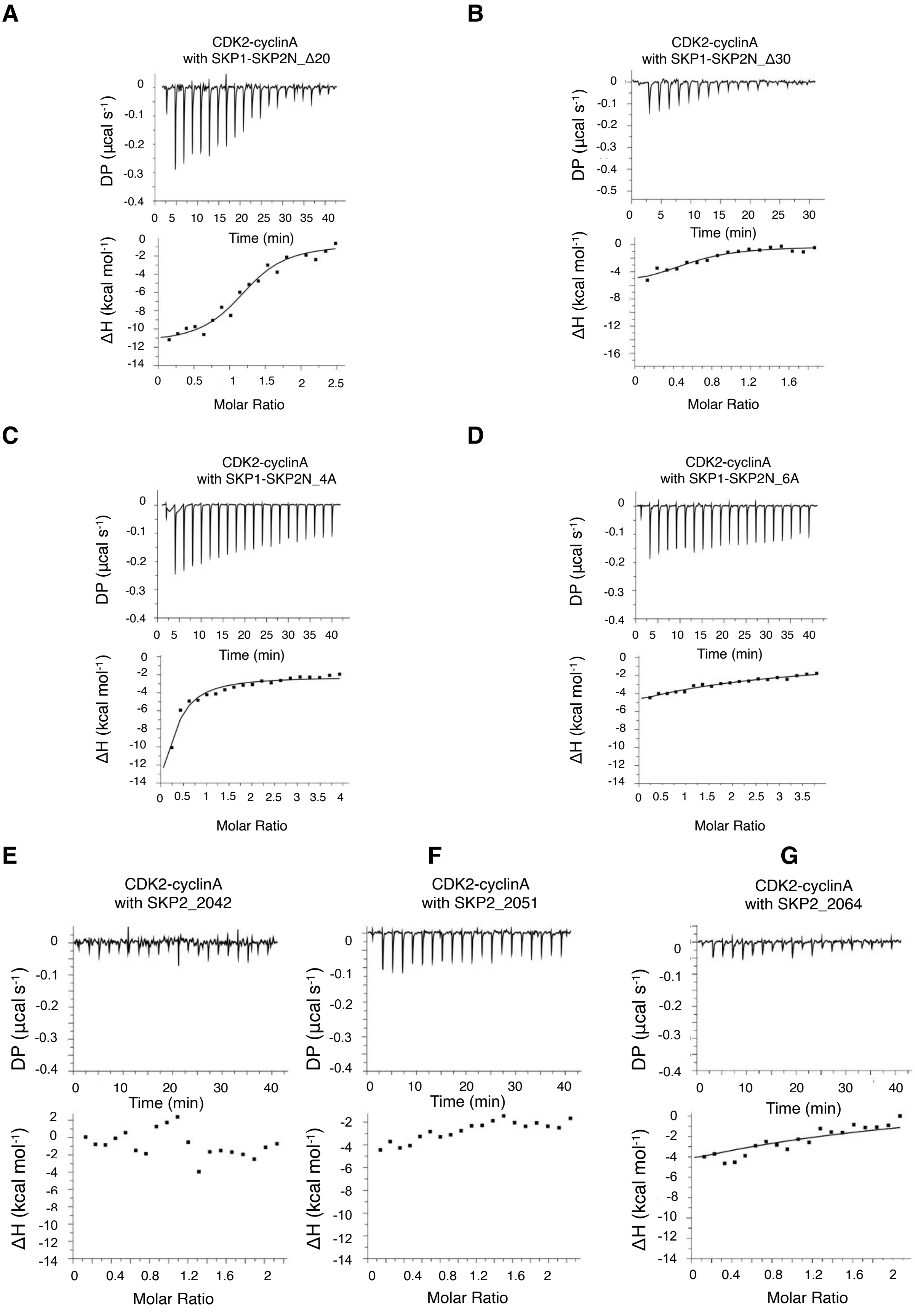
A
CDK2-cyclinA with SKP1-SKP2N_Δ20
B
CDK2-cyclinA with SKP1-SKP2N_Δ30
C
CDK2-cyclinA with SKP1-SKP2N_4A
D
CDK2-cyclinA with SKP1-SKP2N_6A
E
CDK2-cyclinA with SKP2_2042
F
CDK2-cyclinA with SKP2_2051
G
CDK2-cyclinA with SKP2_2064
DP (μcal s-1)
Time (min)
ΔH (kcal mol-1)
Molar Ratio

Supplementary Figure S5. Characterization of the interaction between the SKP2 N-terminal sequence and cyclin A. Related to Table 2. (**A-D**) Isothermal titration calorimetry to characterize the interaction between CDK2-cyclin A and various mutant and truncated SKP2 constructs. (A) SKP1-SKP2N_Δ20, (B) SKP1-SKP2_ Δ30, (C) SKP1-SKP2N_4A (L32A, L33A, S39A, L41A), (D) SKP1-SKP2N_6A (W22A, W24A, L32A, L33A, S39A, L41A). (**E-G**) N-terminal SKP2 peptides 20-42 (E), 20-51, (F) and 20-64 (G). The isothermal titration calorimetry experiments were repeated twice using independently prepared protein samples.

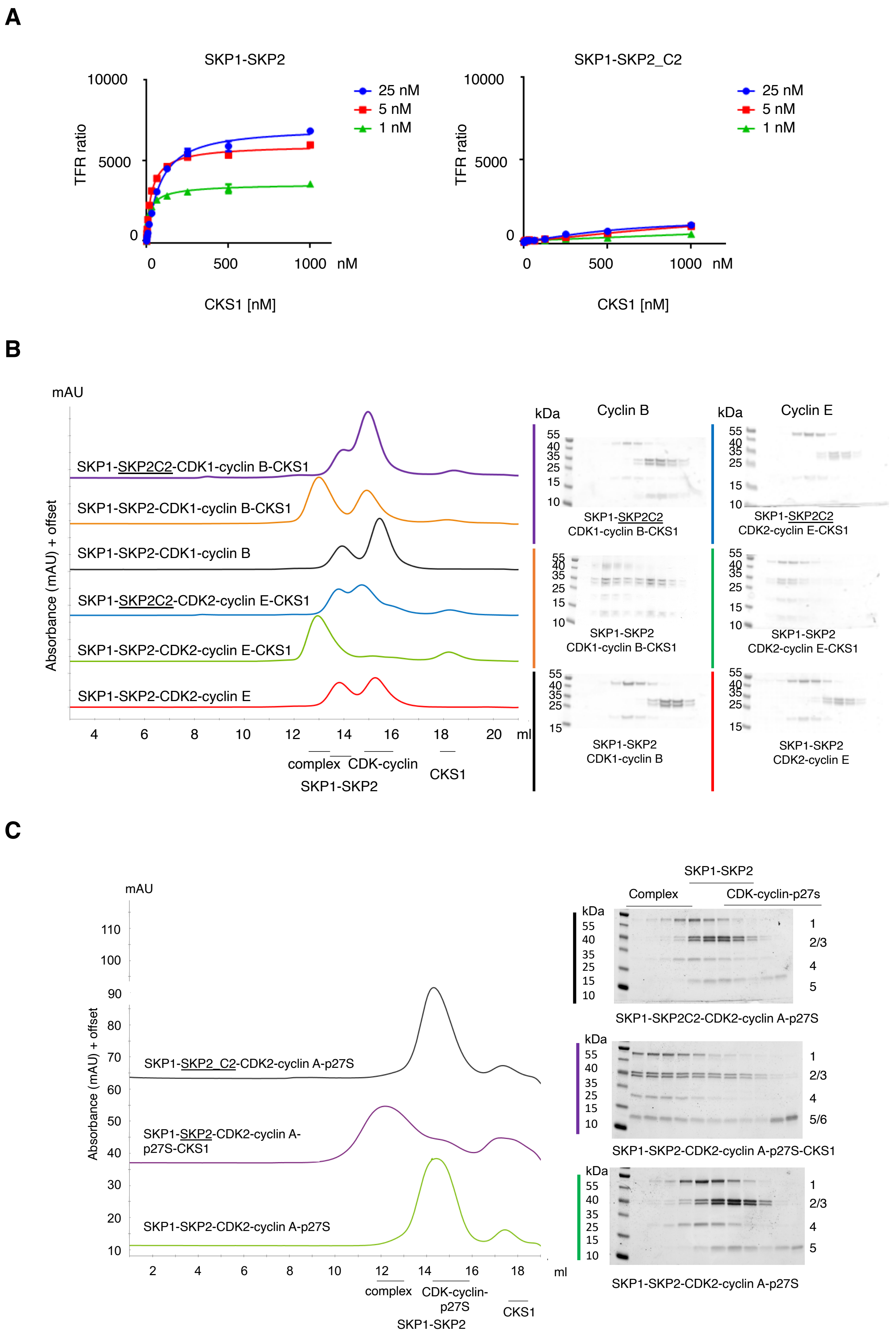
A
SKP1-SKP2
SKP1-SKP2_C2
TFR ratio
10000
5000
0
500
1000 nM
25 nM
5 nM
1 nM
CKS1 [nM]
B
mAU
Absorbance (mAU) + offset
SKP1-SKP2C2-CDK1-cyclin B-CKS1
SKP1-SKP2-CDK1-cyclin B-CKS1
SKP1-SKP2-CDK1-cyclin B
SKP1-SKP2C2-CDK2-cyclin E-CKS1
SKP1-SKP2-CDK2-cyclin E-CKS1
SKP1-SKP2-CDK2-cyclin E
4 6 8 10 12 14 16 18 20 ml
complex CDK-cyclin
SKP1-SKP2
CKS1
kDa
Cyclin B
Cyclin E
55 40 35 25 15 10
SKP1-SKP2C2
CDK1-cyclin B-CKS1
SKP1-SKP2C2
CDK2-cyclin E-CKS1
SKP1-SKP2
CDK1-cyclin B-CKS1
SKP1-SKP2
CDK2-cyclin E-CKS1
SKP1-SKP2
CDK1-cyclin B
SKP1-SKP2
CDK2-cyclin E
C
mAU
Absorbance (mAU) + offset
110 100 90 80 70 60 50 40 30 20 10
SKP1-SKP2_C2-CDK2-cyclin A-p27S
SKP1-SKP2-CDK2-cyclin A-
p27S-CKS1
SKP1-SKP2-CDK2-cyclin A-p27S
2 4 6 8 10 12 14 16 18 ml
complex CDK-cyclin-
p27S
SKP1-SKP2
CKS1
SKP1-SKP2
Complex
CDK-cyclin-p27s
kDa
55 40 35 25 15 10
1
2/3
4
5
5/6
SKP1-SKP2C2-CDK2-cyclin A-p27S
SKP1-SKP2-CDK2-cyclin A-p27S-CKS1
SKP1-SKP2-CDK2-cyclin A-p27S

Supplementary Figure S6. Characterization of the interaction between SKP2 and CKS1. Related to Figure 5.

(**A**) Homogenous-time-resolved fluorescence (HTRF) assay to measure the binding of CKS1 to SKP1-SKP2 (left hand panel) and SKP1-SKP2_C2 (right hand panel). SKP2 (residues 101-436) co-expressed with SKP1 (1-163, Δ38-43 and Δ69-81)), and CKS1 (residues 5-73) are N-terminally tagged with GST or the AviTag respectively. The derived K_d values were dependent on the SKP1-SKP2 concentration (at 25, 5 and 1 nM, equal to 81.0, 31.3 and 20.8 nM respectively) from which it can be deduced that the actual K_d value for the interaction is circa < 1 nM (the lowest SKP1-SKP2 concentration used in the experiment). Replacement of SKP2 F393 with a G-S pair in the construct SKP1-SKP2_C2 severely impairs CKS1 binding to SKP2. The error bars represent the SEM. At least two independent repeats were carried out for each experiment. (**B**) The CDK2-CKS1 or CDK1-CKS1 interface is sufficient to maintain a stable pentameric complex in the absence of a cyclin-SKP2 interaction. Analytical size exclusion chromatography (SEC) assesses the ability of CDK1-cyclin B, CDK2-cyclin A and CDK2-cyclin E to form complexes with SKP1-SKP2 in the presence and absence of CKS1. SKP1, SKP2 and CKS1 are all full-length. Chromatograms represent single experiments and are displayed offset along the y-axis for clarity. (**C**) The interaction of p27 with SKP2 is dependent on CKS1. Analytical SEC shows that p27 bound to CDK2-cyclin A forms a complex with SKP2 in the presence of CKS1 (compare magenta and green traces). When the CKS1 binding site on SKP2 is compromised (by introduction of the SKP2_C2 mutation) CDK2-cyclin A-CKS1 and SKP1-SKP2 complexes are detected (black trace). Chromatograms are representative of 2 biological replicates carried out using either p27M (residues 1-106) or p27S (residues 23-106). Experiment using p27S is shown and chromatograms are displayed offset along the y-axis for clarity. SKP2, CDK2, cyclin A, SKP1, p27S and CKS1 are identified by numbers 1-6 respectively. CKS1 (9.6 kDa) and p27S (9.9 kDa) co-migrate.

Supplementary Table S1. Data collection and refinement statistics. Related to Figure 2.

	CDK2-cyclin Amut5
**Data collection**	
Space group	$P12_11$
Cell dimensions	
a, *b*, *c* (Å)	40.3 137.8 109.7
α, β, γ (°)	90.0 99.8 90.0
Resolution (Å)	85.10 - 2.43 (2.52 - 2.43)
R_{sym}/R_{merge}	0.067 (0.587)
$I/\sigma I$	12.0 (1.3)
Completeness (%)	99.6 (98.7)
Redundancy	1.9 (1.9)
**Refinement**	
Resolution (Å)	85.10 - 2.43 (2.52 - 2.43)
No. reflections	44272 / 2129
R_{work} / R_{free} (%)	20 / 25.1
No. atoms	
Protein	8366
Ligand/ion	22
Water	97
B-factors	
Protein	47.2
Ligand/ion	51.5
Water	32.1
R.m.s. deviations	
Bond lengths (Å)	0.005
Bond angles (°)	1.38

- Structure was determined from a single crystal following screening.
- Values in parentheses are for highest-resolution shell.

Supplementary Table S2. Kinase activity of CDK2-cyclin A complexes. Related to Figure 2. Complexes were assayed using a p107 substrate peptide using the ADP-Glo™ assay format as described in the methods. [1]ND (not determined). [2]Experiments were repeated twice and error bars correspond to the range of values. ATP concentration, 75 µM. [3]Experiments were performed in triplicate and error bars correspond to the range of values. ATP concentration, 25 µM.

	V_{max}	K_m	k_{cat} (1/s)	k_{cat}/K_m (nM/s)
[2]CDK2-cyclin A	5.9 ± 1.2	40.7 ± 17.3	2.4	0.1
[2]CDK2-cyclin A-SKP1-SKP2	7.6 ± 0.5	12.3 ± 2.5	3.1	0.2
[2]CDK2-cyclin A-SKP1-SKP2^{S64A}	6.8 ± 0.7	15.1 ± 4.3	2.7	0.2
[2]SKP1-SKP2^{S64A}	[1] ND	ND	ND	ND
[3]CDK2-cyclin A	19.8 ± 3.4	58.66 ± 19.4	7.9	0.1
[3]CDK2-cyclin A-p27	ND	ND	ND	ND
[3]CDK2-cyclin Amut5	19.5 ± 2.1	34.01 ± 8.5	7.8	0.2
[3]CDK2-cyclin Amut7	21.3 ± 1.2	31.62 ± 4.3	8.5	0.3

Supplementary Table S3. SAXS data collection and processing. Related to Figure 4.

**Data collection**	
Beamline	BL21-Diamond
X-ray wavelength (KeV)	12.4
q range (Å)	0.004-0.4420
Concentration range ($mg.mL^{-1}$)	5 – 0.2
Temperature (^{o}C)	10
**Data Analysis**	
$I_{(0)}$	3.88E-2
Guinier q-region	1-1383
Rg (Å) from Guinier (±SE)	35.32 (± 0.39)
Rg (Å) from GNOM (±SE)	34.86 (± 0.55)
D_{max} (Å)	110
Resolution (Å)	16.1
Oligomeric state	monomer
**Ab Initio modelling**	
Number of models	12
**Software employed**	
Primary data reduction	Scatter3
Theoretical data fitting	Scatter3
Envelope modelling	DAMMIF
Modelling	Coral and EOM

Supplementary References

[1] Brown, N.R., et al., *The structural basis for specificity of substrate and recruitment peptides for cyclin-dependent kinases.* Nat Cell Biol, 1999. **1**: p. 438-443.

[2] Honda, R., et al., *The structure of cyclin E1/CDK2: implications for CDK2 activation and CDK2-independent roles.* EMBO J, 2005. **24**: p. 452-463.

[3] Brown, N.R., et al., *CDK1 structures reveal conserved and unique features of the essential cell cycle CDK.* Nat Commun, 2015. **6**: p. 6769.
